# Control of Anther Cell Differentiation by the Small Protein Ligand TPD1 and Its Receptor EMS1 in *Arabidopsis*

**DOI:** 10.1371/journal.pgen.1006147

**Published:** 2016-08-18

**Authors:** Jian Huang, Tianyu Zhang, Lisa Linstroth, Zachary Tillman, Marisa S. Otegui, Heather A. Owen, Dazhong Zhao

**Affiliations:** 1 Department of Biological Sciences, University of Wisconsin-Milwaukee, Milwaukee, Wisconsin, United States of America; 2 Department of Botany, University of Wisconsin-Madison, Madison, Wisconsin, United States of America; INRA, FRANCE

## Abstract

A fundamental feature of sexual reproduction in plants and animals is the specification of reproductive cells that conduct meiosis to form gametes, and the associated somatic cells that provide nutrition and developmental cues to ensure successful gamete production. The anther, which is the male reproductive organ in seed plants, produces reproductive microsporocytes (pollen mother cells) and surrounding somatic cells. The microsporocytes yield pollen via meiosis, and the somatic cells, particularly the tapetum, are required for the normal development of pollen. It is not known how the reproductive cells affect the differentiation of these somatic cells, and vice versa. Here, we use molecular genetics, cell biological, and biochemical approaches to demonstrate that TPD1 (TAPETUM DETERMINANT1) is a small secreted cysteine-rich protein ligand that interacts with the LRR (Leucine-Rich Repeat) domain of the EMS1 (EXCESS MICROSPOROCYTES1) receptor kinase at two sites. Analyses of the expressions and localizations of TPD1 and EMS1, ectopic expression of TPD1, experimental missorting of TPD1, and ablation of microsporocytes yielded results suggesting that the precursors of microsporocyte/microsporocyte-derived TPD1 and pre-tapetal-cell-localized EMS1 initially promote the periclinal division of secondary parietal cells and then determine one of the two daughter cells as a functional tapetal cell. Our results also indicate that tapetal cells suppress microsporocyte proliferation. Collectively, our findings show that tapetal cell differentiation requires reproductive-cell-secreted TPD1, illuminating a novel mechanism whereby signals from reproductive cells determine somatic cell fate in plant sexual reproduction.

## Author Summary

The differentiation of distinct somatic and reproductive cells in flowers is required for the successful sexual reproduction of plants. The anther produces reproductive microsporocytes (pollen mother cells) that give rise to pollen (male gametophytes), as well as surrounding somatic cells (particularly the tapetal cells) that support the normal development of pollen. In animals, signals from somatic cells are known to influence reproductive cell fate determination, and vice versa. However, little is known about the molecular mechanisms underlying somatic and reproductive cell fate determination in plants. In this paper, we demonstrate that TPD1 (TAPETUM DETERMINANT1) is processed into a small secreted cysteine-rich protein ligand for the EMS1 (EXCESS MICROSPOROCYTES1) leucine-rich repeat receptor-like kinase (LRR-RLK). TPD1 is secreted from reproductive cells to the plasma membrane of somatic cells, where activated TPD1-EMS1 signaling first promotes periclinal cell division and then determines tapetal cell fate. Moreover, tapetal cells suppress microsporocyte proliferation. Our findings illuminate a novel mechanism by which reproductive cells determine somatic cell fate, and somatic cells in turn limit reproductive cell proliferation. Plants extensively employ LRR-RLKs to control growth, development, and defense. Our identification of TPD1 as the first small protein ligand for all LRR-RLKs characterized to date will provide a valuable system for studying how small protein ligands activate LRR-RLK signaling complexes.

## Introduction

Successful sexual reproduction depends on the specification of different types of somatic and reproductive cells that give rise to eggs and sperm in both plants and animals. The anther is where male gametophytes (pollen) are produced in seed plants; it typically has four lobes (microsporangia) organized into two thecae, each of which has one abaxial and one adaxial lobe [1–6]. Within each anther lobe, the central reproductive microsporocytes (or pollen mother cells) are surrounded by four concentrically organized somatic cell layers: the epidermis, endothecium, middle layer, and tapetum (listed from outside to inside). Microsporocytes yield pollen via meiosis, and the somatic cells, particularly the tapetal cells, are essential for the normal development and release of pollen. Microsporocytes produce tetrads, each of which contains four haploid spores that later mature into pollen. The tapetum is required to remodel the callose coat surrounding microsporocytes and tetrads, provide nutritive support of microsporocytes and pollen, and synthesize most components of the pollen wall. All anther cells originate from Layer 1 (L1), L2, and L3 cells. The L1 cells form the epidermis, while the L3 cells contribute to forming the vascular column found at the center of each anther and the connective tissue that links the lobes to the vasculature [1–3,5,7–10]. Within an anther lobe, all cells except for those of the epidermis trace back to L2 meristem cells, which give rise to archesporial cells (AR) and subepidermal L2 cells. AR form sporogenous cells and eventually differentiate into microsporocytes. The subepidermal L2 cells differentiate into primary parietal cells (PPC), which undergo periclinal division to produce two layers of secondary parietal cells (SPC). The outer SPC (OSPC) form the endothecium adjacent to the epidermis, and the inner SPC (ISPC) undergo a further periclinal division to establish the middle layer and tapetum, which completes the cell fate specification events in the anther lobe. As the anther is centrally important for plant sexual reproduction and breeding, it is imperative that we obtain an in-depth understanding of the molecular mechanisms underlying somatic and reproductive cell differentiation during anther development.

In *Arabidopsis*, the EMS1 (EXCESS MICROSPOROCYTES1) leucine-rich repeat receptor-like kinase (LRR-RLK) and the small protein, TPD1 (TAPETUM DETERMINANT1), play vital roles in anther cell differentiation. Anthers in *ems1* [also known as *exs* (*extra sporogenous cells*)] and *tpd1* mutants lack tapetal cells but produce excess microsporocytes, which normally enter meiosis [10–12]. In rice, the *MSP1* (*MULTIPLE SPOROCYTE 1*) gene encodes an EMS1-like protein, and the TPD1 orthologs, MAC1 (MULTIPLE ARCHESPORIAL CELLS1) and TDL1A [also known as MIL2 (MICROSPORELESS2)], have been identified in maize and rice, respectively [13–16]. Disruption of the *MSP1*, *MIL2/TDL1A*, and *MAC1* genes yield anther phenotypes similar to those of *ems1* and *tpd1* mutants [13–17]. Elegant studies in maize showed that, in response to hypoxia, the glutaredoxin, MSCA1 (MALE STERILE CONVERTED ANTHER1), activates AR differentiation [18] likely through the TGA transcription factors [19]. Once the AR are specified, AR-derived MAC1 proteins directly induce the neighboring subepidermal L2 cells to differentiate into PPC while also autonomously limiting the proliferation of AR and subepidermal L2 cells [15,18]. Previous studies have suggested models for the functions of the TPD1/MAC1/MIL2-EMS1/MSP1 (for simplicity, only TPD1-EMS1 is used hereafter) signaling system in anther cell differentiation. There is, however, some debate among these models. In tapetal cell differentiation, some reports have proposed that TPD1-EMS1 determines tapetal cell fate [10,15], while others have found that it does not determine tapetal cell fate, but instead exclusively stimulates a small group of tapetal founder cells to proliferate into a monolayer of tapetum [20]. Moreover, in microsporocyte proliferation, some authors have hypothesized that TPD1-EMS1 limits the proliferation of archesporial cells [11,15,18], while others believe that it suppresses surrounding parietal cells, causing them to “transdifferentiate” into microsporocytes [10,13]. Thus, it is not yet clear how TPD1-EMS1 controls tapetal cell differentiation and microsporocyte proliferation.

Although LRR-RLKs are known to be involved in a wide range of plant growth, developmental, physiological, and defensive processes, only a few ligands have been identified for them. These include small post-translationally modified peptides and cysteine-rich peptides as two major types of oligopeptide ligands for LRR-RLKs [21], and the hormone ligand, brassinosteroid (BR), which activates BRASSINOSTEROID INSENSITIVE1 (BRI1), BRI1-LIKE1 (BRL1), and BRL3 [22]. The small (usually less than 20 residues) post-translationally modified peptides include: CLAVATA3 (CLV3), which is a 12-residue peptide ligand for CLAVATA1 (CLV1) [23,24]; PHYTOSULFOKINE 1 (PSK1), which is a 5-residue peptide ligand for PSKR1 [25]; tracheary element differentiation inhibitory factor (TDIF), which is a 12-residue peptide ligand for TDIF RECEPTOR (TDR) [26,27]; INFLORESCENCE DEFICIENT IN ABSCISSION (IDA), which is a 12-residue peptide ligand for HAESA (HAS) and HAESA-LIKE2 (HSL2) [28,29]; and GRIM REAPER (GRI), which is an 11-residue peptide ligand for POLLEN-SPECIFIC RECEPTOR-LIKE KINASE 5 (PRK5) [30].

The cysteine-rich peptides usually contain a C-terminal domain with 4–16 cysteine residues [21]. EPIDERMAL PATTERNING FACTORS 2 (EPF2) and STOMAGEN (EPFL9) serve as ligands for competitively binding ERECTA (ER)-family LRR-RLKs [31–33]. LAT52 and STIGMA-SPECIFIC PROTEIN1 (STIG1, a processed 7-kD peptide) act as ligands of the pollen-specific receptor kinase, LePRK2 [34,35]. In *Arabidopsis*, TPD1 interacts with EMS1 *in vitro* and *in vivo* [9]. Among the orthologs of TPD1, immunostaining showed that the maize MAC1 protein is enriched in AR cells [15]. MAC1 was observed in the extracellular space of onion cells in transient assays, suggesting that it is secreted, and Western blotting suggested that MAC1 is not further cleaved besides the removal of the putative signal peptide. So far, however, the receptor of MAC1 has not been identified. Finally, Bimolecular Fluorescence Complementation (BiFC) assay showed that the rice TDL1A interacts with MSP1 in onion-cell cytoplasm [14]. These data suggested that the presumptive ligand, TPD1/MAC1/TDL1A, might be the first identified small protein ligand for an LRR-RLK. However, several key points had not been established biochemically in previous studies.

Here, we demonstrate for the first time that TPD1 is a 13-kD small secreted cysteine-rich protein ligand that interacts with the EMS1 LRR domain at two sites. TPD1-EMS1 signaling initially promotes the periclinal division of secondary parietal cells to form a monolayer of tapetal cell precursors, and then determines the fate of functional tapetal cells. Our findings that tapetal cell differentiation requires TPD1 secreted from normal pre-meiotic cells illuminate a novel mechanism by which signals from reproductive cells determine somatic cell fate in plant sexual reproduction.

## Results

### TPD1 is processed at its C-terminus to generate a 13-kD small cysteine-rich protein

**Fig 1:**
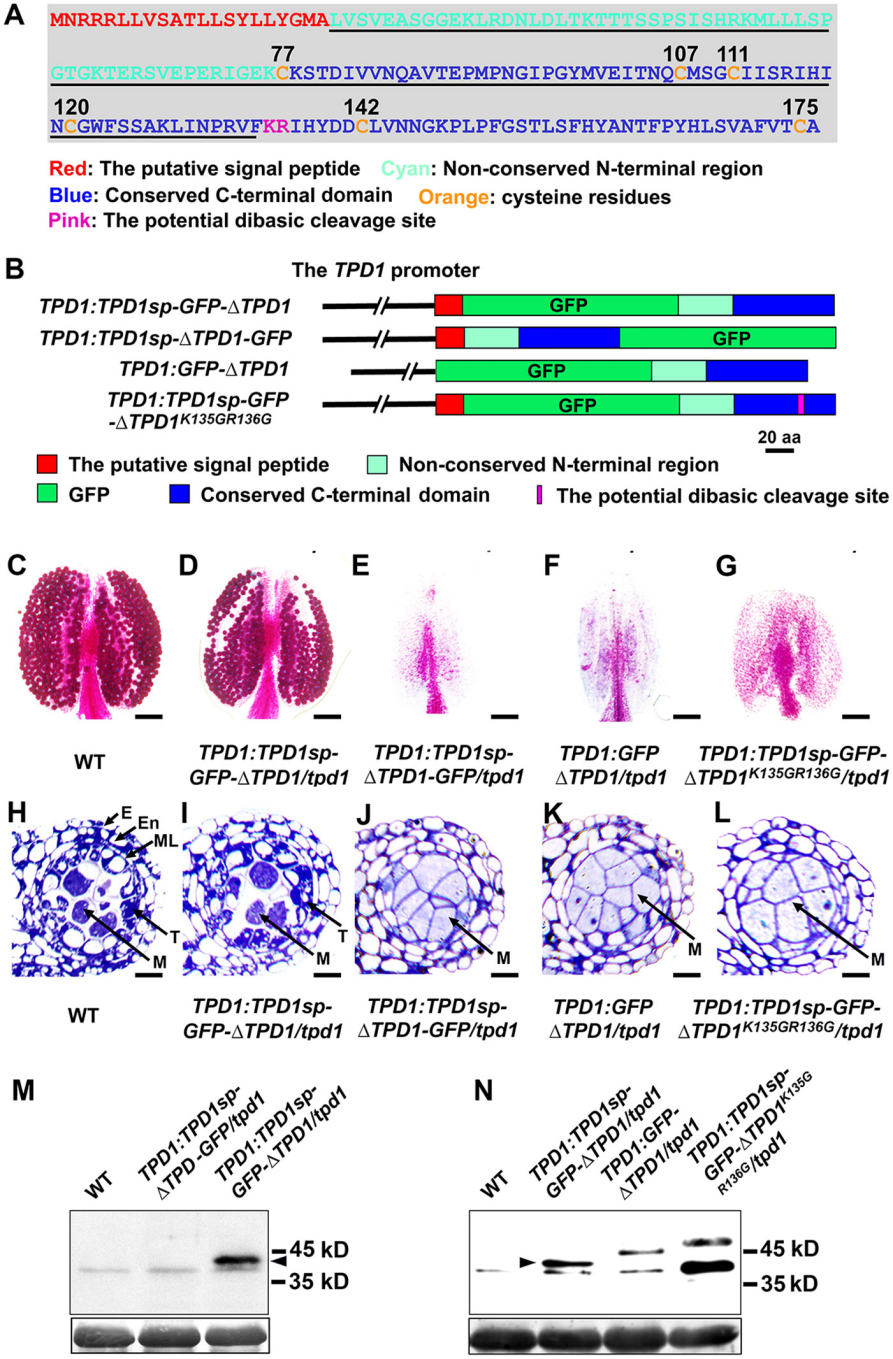
TPD1 is processed into a 13-kD small protein in planta. (**A**) The TPD1 protein sequence. Red color: the putative signal peptide, Cyan: the non-conserved N-terminal region, Blue: the conserved C-terminal domain, Orange: cysteine residues (numbers indicate their positions), Pink “KR”: the potential dibasic cleavage site, and Underlined: the sequence of identified mature TPD1. (**B**) To examine the processing of TPD1, constructs encoding GFP-TPD1 fusion proteins were generated and introduced into the *tpd1* mutant. Schematic diagrams showing the constructs used for the complementation experiments. The 2.7-kb *TPD1* promoter was used in all constructs. Red bar: the putative signal peptide (Sp) of TPD1, Green bar: GFP, Cyan bar: the non-conserved N-terminal region, Blue bar: the conserved C-terminal domain, ΔTPD1: TPD1 without the putative signal peptide, and Pink line: K135GR135G mutations. All constructs were transformed into *tpd1* heterozygous plants. PCR genotyping was carried out to determine the *tpd1* mutant background of T2 transgenic plants. Transgenic plants showing siliques comparable in size to those of wild-type plants were counted as complemented plants. (**C**-**G**) Alexander pollen staining of mature anthers reveals viable pollen grains in wild-type (**C**) and *TPD1*:*TPD1sp-GFP-ΔTPD1/tpd1* (**D**) plants, whereas no pollen is observed in *TPD1*:*TPD1sp-ΔTPD -GFP/tpd1* (**E**), *TPD1*:*GFP-ΔTPD1/tpd1* (**F**), and *TPD1*:*TPD1sp-GFP-ΔTPD1* ^*K135G R136G*^/*tpd1* (**G**) anthers. Scale bars, 50 μm. (**H**-**L**) Semi-thin sections of stage-6 anthers show normal tapetal cells and microsporocytes in wild-type (**H**) and *TPD1*:*TPD1sp-GFP-ΔTPD1/tpd1* (**I**) plants, whereas a complete lack of tapetal cells and the presence of excess microsporocytes are seen in *TPD1*:*TPD1sp-ΔTPD -GFP/tpd1* (**J**), *TPD1*:*GFP-ΔTPD1/tpd1* (**K**), and *TPD1*:*TPD1sp-GFP-ΔTPD1* ^*K135G R136G*^/*tpd1* (**L**) anthers. E, epidermis; En, endothecium; M, microsporocyte; ML, middle layer; and T, tapetal cell. Scale bars, 10 μm. (**M** and **N**) Western blot analysis of the processing of GFP-fused TPD1 proteins. (**M**) No band was seen for wild-type (WT) or *TPD1*:*TPD1sp-ΔTPD -GFP/tpd1* plants, whereas *TPD1*:*TPD1sp-GFP-ΔTPD1/tpd1* plants exhibited a 41-kD band. By subtracting 28 kD for GFP, we calculate that the mature TPD1 protein is about 13 kD in size. (**N**) Wild-type (WT) plants showed no band; *TPD1*:*TPD1sp-GFP-ΔTPD1/tpd1* plants exhibited a 41-kD band; *TPD1*:*GFP-ΔTPD1/tpd1* plants exhibited a non-cleaved 45-kD band; and *TPD1*:*TPD1sp-GFP-ΔTPD1*^*K135G R136G*^*/tpd1* plants exhibited a non-cleaved 48-kD band. Arrowheads indicate the 41-kD bands. Bottom: Coomassie blue staining of RuBisCO.

The *TPD1* gene encodes a 176-residue small protein (19.5 kD) that contains a putative 21-residue signal peptide at the N-terminus, a non-conserved N-terminal region, and a conserved cysteine-rich C-terminal domain (Fig 1A and S1 Fig). Our sequence analysis revealed that the TPD1 C-terminal domain contains the dibasic site, K135R136 (Fig 1A and S1 Fig), which is a conserved proteolytic cleavage site for precursor peptide processing in animals and yeast [36]. To investigate whether TPD1 is similarly processed in planta, we generated *TPD1*:*TPD1sp-GFP-ΔTPD1* (ΔTPD1: full-length TPD1 without the putative signal peptide) and *TPD1*:*TPD1sp-ΔTPD1-GFP* transgenic plants to produce TPD1 N-terminal and C-terminal GFP fusion proteins (Fig 1B). Compared with wild-type plants (Fig 1C and 1H), 71.6% (68/95) of *TPD1*:*TPD1sp-GFP-ΔTPD1*/*tpd1* plants produced normal pollen and anthers (Fig 1D and 1I), indicating complementation, whereas *TPD1*:*TPD1sp-ΔTPD1-GFP* (0/42) failed to complement the *tpd1* phenotype (Fig 1E and 1J). Therefore, the TPD1 N-terminal GFP fusion protein had a function comparable to that of the native TPD1, but TPD1 fused with GFP at its C-terminus exhibited functional impairment.

To test if the putative signal peptide affects the processing of TPD1 and whether TPD1 is cleaved at the predicted K135R136 dibasic site, we generated *TPD1*:*GFP-ΔTPD1* and *TPD1*:*TPD1sp-GFP-ΔTPD1* ^*K135G R136G*^ transgenic plants to produce a TPD1 N-terminal GFP fusion protein without the signal peptide (GFP-ΔTPD1) and a TPD1sp-GFP-ΔTPD1 ^K135G R136G^ protein containing mutations in the K135R136 dibasic site (Fig 1B). However, neither *TPD1*:*GFP-ΔTPD1* (Fig 1F and 1K) nor *TPD1*:*TPD1sp-GFP-ΔTPD1* ^*K135G R136G*^ (Fig 1G and 1L) complemented the *tpd1* phenotype.

When proteins extracted from young buds were subjected to Western blot analysis with an antibody against GFP, we did not detect any signal from the wild-type (Fig 1M, Lane 1 and Fig 1N, Lane 1) or *TPD1*:*TPD1sp-ΔTPD1-GFP/tpd1* (Fig 1M; Lane 2) samples, but we did observe a 41-kD band in the *TPD1*:*TPD1sp-GFP-ΔTPD1*/*tpd1* sample (Fig 1M, Lane 3 and Fig 1N; Lane 2). We also detected 45-kD and 48-kD bands corresponding to the predicted sizes of the fusion proteins expressed in *TPD1*:*GFP-ΔTPD1*/*tpd1* (Fig 1N; Lane 3) and *TPD1*:*TPD1sp-GFP-ΔTPD1*^*K135G R136G*^ /*tpd1* (Fig 1N; Lane 4) plants, respectively, indicating that ΔTPD1-GFP and TPD1sp-GFP-ΔTPD1^K135G R136G^ did not undergo cleavage.

Similar results were obtained using the leaf protoplast transient expression system and Western blotting. *35S*:*TPD1sp-GFP-ΔTPD1*, *35S*:*GFP-ΔTPD1*, and *35S*:*TPD1sp-GFP-ΔTPD1*^*K135G R136G*^ were introduced into leaf protoplasts obtained from *35S*:*EMS1* plants (S2 Fig). The 41-kD band was detected in the *35S*:*TPD1sp-GFP-ΔTPD1* sample, whereas 45-kD and 48-kD bands were found in *35S*:*TPD1sp-GFP-ΔTPD1* and *35S*:*TPD1sp-GFP-ΔTPD1*^*K135G R136G*^ samples, respectively (S2 Fig).

The 41-kD band detected in *TPD1*:*TPD1sp-GFP-ΔTPD1*/*tpd1*-complemented plants and *35S*:*TPD1sp-GFP-ΔTPD1 35S*:*EMS1* protoplasts is smaller than the 45 kD predicted for the full-length fusion protein without the putative signal peptide or the 48-kD predicted for the full-length fusion protein. Considering that GFP is 28 kD in size, our results suggest that cleavage occurs around the C-terminal dibasic site, K135R136, yielding an approximately 13-kD TPD1 protein (residues 22 to possibly 134) that includes four cysteines (Fig 1A). In addition, our findings indicate that the putative signal peptide is important for TPD1 cleavage.

**Fig 2:**
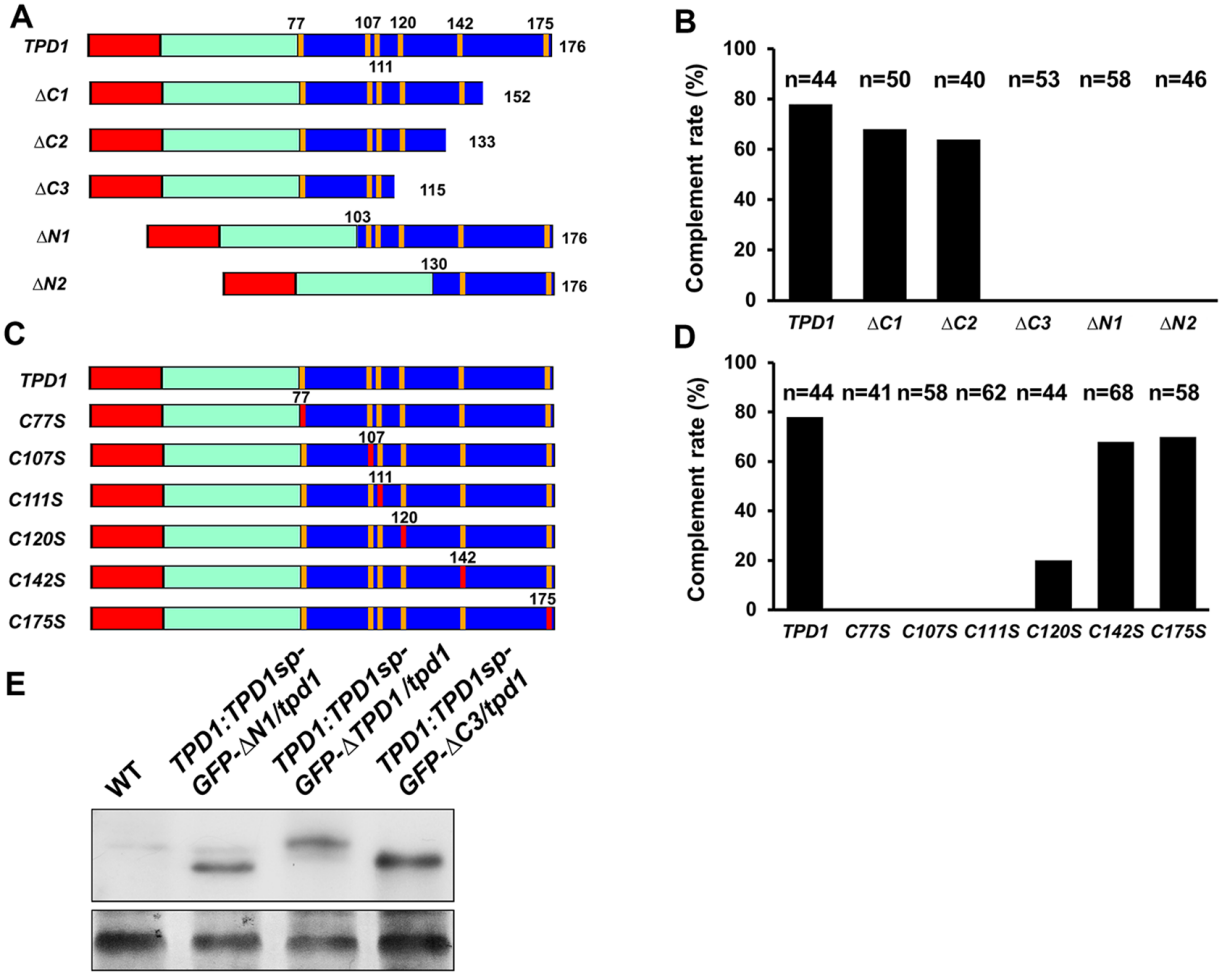
Genetic identification of the mature TPD1 protein and importance of cysteine residues. Red bar: the TPD1 putative signal peptide, Cyan bar: the non-conserved N-terminal region, and Blue bar: the conserved C-terminal domain, with cysteine positions shown as vertical orange lines. The 2.7-kb *TPD1* promoter was used in all constructs, which were introduced into the *tpd1* mutant. The complementation rate (%) indicates the percentage of complemented plants among all examined plants. Plants with normal siliques were counted as complemented plants. (A) Schematic diagrams showing the full-length *TPD1* and a series of truncations from the N- and C-terminal ends of the conserved C-terminal domain. Numbers indicate amino acid positions. (B) Complementation rates (%) of the *TPD1* truncations. Compared with the full-length *TPD1*, Δ*C1* and Δ*C2* rescued the *tpd1* phenotype, but Δ*C3*, Δ*N1* and Δ*N2* did not (n = numbers of examined plants). (C) Schematic diagrams showing *TPD1* constructs with mutated cysteine residues. Red vertical lines indicate substitutions of cysteines to serines. (D) Complementation rates (%) of site-mutated versions of *TPD1* (mutations on cysteine residues: *C77S*, *C107S*, *C111S*, *C120S*, *C142S*, and *C175S*; n = numbers of examined plants). *C77S*, *C107S*, and *C111S* failed to complement the *tpd1* phenotype; *C120S* exhibited a small complementation effect; and *C142S* and *C175S* complemented the *tpd1* phenotype. (E) Western blotting was used to confirm the expressions of the truncated ΔN1 and ΔC3 proteins. Wild-type (WT) plants exhibited no band; *TPD1*:*TPD1sp-GFP-ΔN1/tpd1* plants exhibited a 38-kD band; *TPD1*:*TPD1sp-GFP-ΔTPD1/tpd1* plants exhibited a 41-kD band; and *TPD1*:*TPD1sp-GFP-ΔC3/tpd1* plants exhibited a 39-kD band. Bottom: Coomassie blue staining of RuBisCO.

To test the functionality of the processed TPD1, we performed genetic complementation experiments by generating a series of N-terminal and C-terminal truncations of the conserved C-terminal domain of TPD1 (Fig 2A). We examined at least 40 independent transgenic lines for each truncation, assessing their ability to restore pollen fertility in the *tpd1* mutant background (Fig 2B). Same as the full-length *TPD1* gene and *ΔC1*, a short C-terminal *TPD1* truncation version *ΔC2*, which encodes the processed TPD1 protein, complemented the *tpd1* phenotype (Fig 2B). However, the further C-terminal *TPD1* truncation mutant, *ΔC3*, did not complement the *tpd1* phenotype. Moreover, *ΔN1* and *ΔN2*, which encoded only short C-terminal domains, failed to complement the *tpd1* phenotype (Fig 2A and 2B). These results rule out the possibility that TPD1 is processed into a small functional peptide that contains only the far end of its C-terminus. To confirm that the non-complementing constructs yielded actual expression of truncated TPD1 proteins, we generated *TPD1*:*TPD1sp-GFP-ΔN1/tpd1* (n = 36) and *TPD1*:*TPD1sp-GFP-ΔC3/tpd1* (n = 41) transgenic plants. Western blot analysis detected bands of the predicted sizes (38 kD and 39 kD, respectively) (Fig 2E), supporting our contention that truncated TPD1 proteins smaller than the determined mature size were not functional.

The C-terminal domain of TPD1 has six cysteine residues (C77, C107, C111, C120, C142, and C175) (Figs 1A and 2C). To define which residues are essential for the normal function of TPD1, we performed single mutations to replace these cysteines with serines (Fig 2C). Complementation experiments demonstrated that C77, C107, C111 and C120, which lie in the mature TPD1, were required for *TPD1* function, whereas mutations of C142 and C175, which are excluded from the mature TPD1, did not affect *TPD1* function (Fig 2D).

In summary, our results suggest that the processed 13-kD form of TPD1 (residues 22 to possibly 134) is a functional mature small protein ligand that contains four essential cysteines.

### The mature TPD1 interacts with the EMS1 LRR domain at two sites

**Fig 3:**
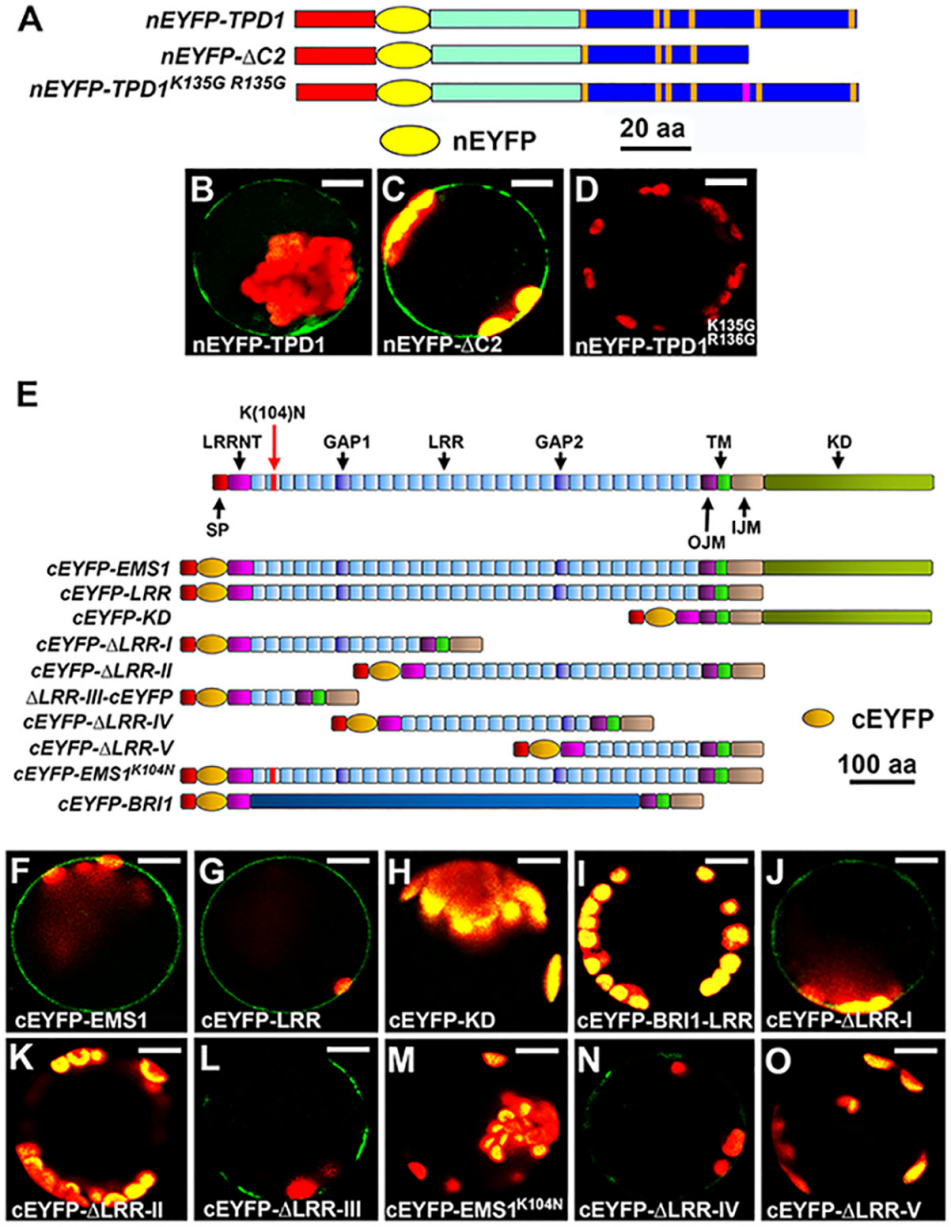
The mature TPD1 interacts with the EMS1 LRR domain at two sites. Bimolecular Fluorescence Complementation (BiFC) experiments were performed using *Arabidopsis* mesophyll protoplasts to test the interaction between the mature TPD1 and EMS1 and determine the TPD1-interacting site(s) in EMS1. *35S*:*EYFP* was used as a control to monitor the transfection efficiency. At least three independent experiments were performed for each assay. (**A**) Schematic diagrams showing the truncated and mutated versions of TPD1. Red bar: the putative signal peptide of TPD1, Cyan bar: the non-conserved N-terminal region, Blue bar: the conserved C-terminal domain, with cysteine positions shown as vertical orange lines, Yellow oval: nEYFP (N-terminal EYFP) inserted right after the signal peptide of TPD1, and Pink line in the *nEYFP- TPD1*^*K135G R136G*^ construct: K135GR136G mutations. Size bar indicates TPD1 alone, and does not include nEYFP. (**B**-**D**) Confocal images showing that cEYFP-LRR (the full-length EMS1 LRR domain) interacts with nEYFP-TPD1 (**B**) and nEYFP-ΔC2 (**C**), but not nEYFP- TPD1^K135G R136G^ (**D**). Scale bars, 10 μm. (**E**-**O**) Identification of TPD1-binding domains in EMS1. (**E**) Schematic diagrams showing the primary structures of EMS1 and the truncated/mutated versions used for the BiFC assays. SP: signal peptide, LRRNT: leucine-rich repeat containing N-terminus, LRR: leucine-rich repeat, GAP: non-leucine-rich repeat gap region between the two LRRs, OJM: outer juxtamembrane domain, IJM: inner juxtamembrane domain, TM: transmembrane domain, KD: kinase domain, Red bar in the cEYFP-EMS1^K104N^ construct: K104N mutation. All EMS1 constructs contained SP, OJM, TM, and IJM. Orange oval represents the C-terminal EYFP (cEYFP). Size bar indicates EMS1 alone, and does not include cEYFP. (**F**-**O**) Confocal images showing that the mature TPD1 (nEYFP-ΔC2) interacts with cEYFP-EMS1 [the full-length EMS1, (**F**)], cEYFP-LRR [the full-length EMS1 LRR domain, (**G**)], cEYFP-ΔLRR-I (**J**), cEYFP-ΔLRR-III (**L**), and cEYFP-ΔLRR-IV (**N**), but not with cEYFP-KD (**H**), cEYFP-BRI1 [(**I)**, negative control], cEYFP-ΔLRR-II (**K**), cEYFP-EMS1^K104N^ (**M**), or cEYFP-ΔLRR-V (**O**). Scale bars, 10 μm.

To determine whether the processed TPD1 interacts with EMS1, we performed Bimolecular Fluorescence Complementation (BiFC) using *Arabidopsis* mesophyll protoplasts. By inserting *nEYFP* (N-terminal EYFP) after the putative signal peptide of TPD1, we generated constructs that produced full-length TPD1 (*nEYFP-TPD1*), mature TPD1 (*nEYFP-ΔC2*), and mutated TPD1 (*nEYFP-TPD1*^*K135G R136G*^) (Fig 3A). We co-transfected each construct plus a *cEYFP* (*C-terminal EYFP*)*-LRR* (includes the full-length LRR domain; Fig 3E)-encoding construct into *Arabidopsis* protoplasts, and then tested for protein interactions at the plasma membrane. Our results showed that full-length TPD1 (Fig 3B) and mature TPD1 (ΔC2) (Fig 3C) both interacted with the EMS1 LRR, but TPD1^K135G R136G^ did not (Fig 3D). This indicates that mature TPD1 (ΔC2) can interact with EMS1, and cleavage processing is essential for the TPD1-EMS1 interaction at the plasma membrane.

To identify where the mature TPD1 interacts with EMS1, we referred to the primary structure of EMS1 and generated a series of EMS1 LRR truncations fused to cEYFP (C-terminal EYFP) (Fig 3E). The *nEYFP-ΔC2* (Fig 3A) construct was used to test for interactions between mature TPD1 and truncated EMS1 LRRs (Fig 3E). The full-length EMS1 (cEYFP-EMS1; Fig 3E and 3F) and the full-length EMS1 LRR domain (cEYFP-LRR; Fig 3E and 3G), but not the EMS1 cytoplasmic kinase domain (cEYFP-KD; Fig 3E and 3H), interacted with the mature TPD1 at the plasma membrane. No interaction was detected between the mature TPD1 and cEYFP-BRI1-LRR (Fig 3E and 3I), indicating the specificity of the TPD1-EMS1 interaction.

We further tested the EMS1 LRR domain by separating it to two parts and generated *cEYFP-ΔLRR-I* and *cEYFP-ΔLRR-II* constructs (Fig 3E). We detected an interaction between TPD1 and cEYFP-ΔLRR-I (Fig 3J), but not between TPD1and cEYFP-ΔLRR-II (Fig 3K). Within the LRR-I truncation, we further determined that the first three LRRs (cEYFP-ΔLRR-III) were sufficient to interact with TPD1 (Fig 3E and 3L). Moreover, the site-directed mutation of K104N in LRR2 (the genetic allele *exs-2*), which causes a phenotype similar to that of the null mutant, *ems1* [10,11], impaired the TPD1-EMS1 interaction (Fig 3E and 3M).

Using the LRR-II truncation, we also detected a weak interaction between cEYFP-ΔLRR-IV and TPD1 (Fig 3E and 3N), but no interaction was observed between cEYFP-ΔLRR-V and TPD1 (Fig 3E and 3O). Our previously identified TPD1 interaction region (TIR) [9] lies in cEYFP-ΔLRR-IV.

Although ectopic expression of *TPD1* (*35S*:*TPD1*) reportedly caused anther development defects and wide siliques [37], our *35S*:*EMS1* plants had no visible phenotype (S3 Fig). The vast majority of *35S*:*TPD1 35S*:*EMS1* plants (87.5%; 35/40) exhibited significant defects, including short stature and twisted leaves, stems, and inflorescences (S3 Fig). In contrast, almost all *35S*:*TPD1 35S*:*EMS1*^*K104N*^ plants (95%; 57/60) resembled *35S*:*TPD1* plants (S3 Fig), indicating that the function of TPD1/EMS1 signaling depends on the ability of TPD1 to interact with EMS1 in the first three LRRs.

Taken together, our results suggest that the mature TPD1 interacts with the EMS1 LRR domain at two sites, and that this is required for the normal function of TPD1.

### The N-terminal signal peptide is essential for the normal function of TPD1 in anther cell differentiation

**Fig 4:**
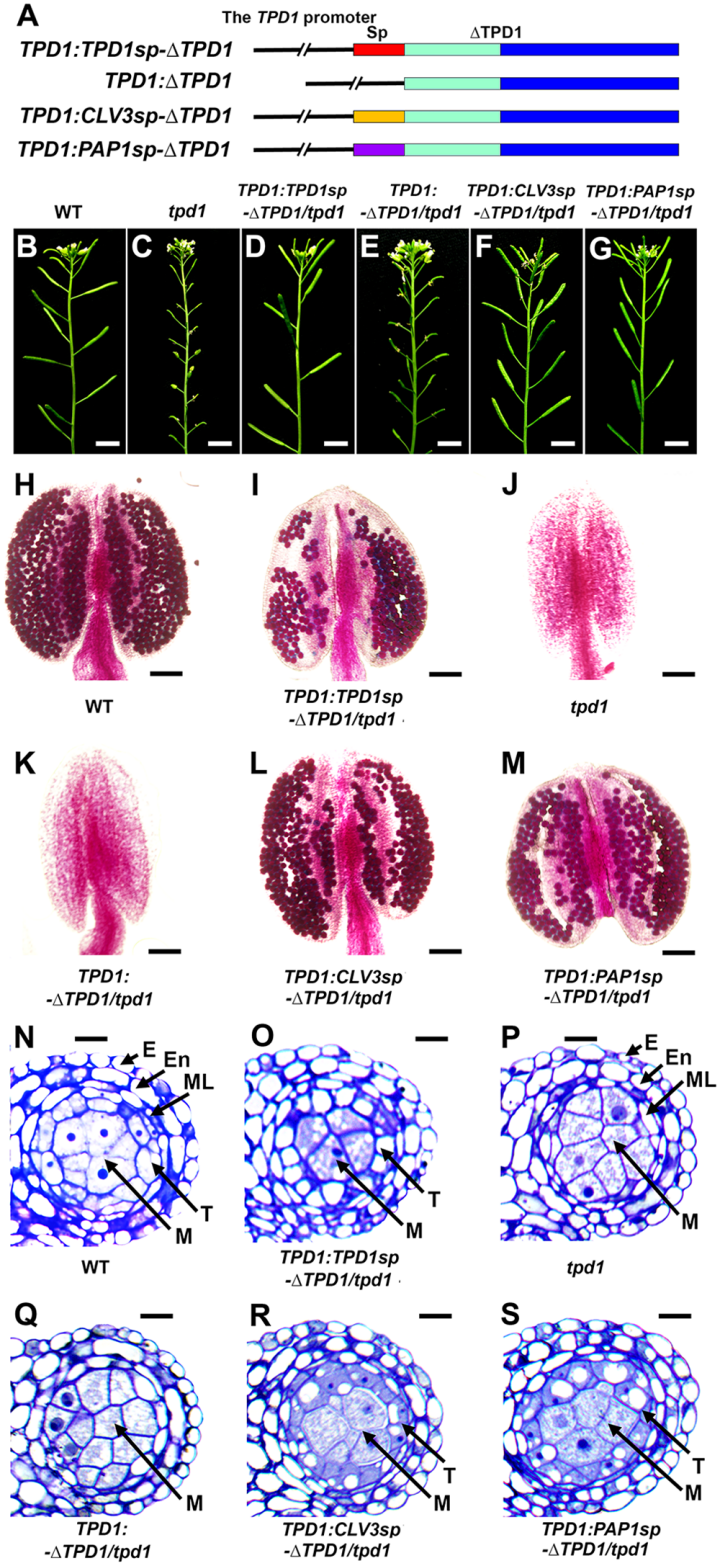
The putative signal peptide of TPD1 is required for its normal function. To test if the putative signal peptide of TPD1 and the N-terminal signal peptide-directed secretion of TPD1 are required for its function in anther cell fate determination, vectors encoding TPD1s in which the putative signal peptide had been replaced with known secreted signal peptides were introduced into the *tpd1* mutant and assessed for their ability to rescue the *tpd1* phenotype. (**A**) Schematic diagrams showing the structures of the *TPD1*:*TPD1sp-*Δ*TPD1*, *TPD1*:Δ*TPD1*, *TPD1*:*CLV3sp-*Δ*TPD1*, and *TPD1*:*PAP1sp-*Δ*TPD1* constructs. Red bar: the TPD1 putative signal peptide, Orange bar: the CLV3 signal peptide, Purple bar: the PAP1 signal peptide, Cyan bar: the non-conserved N-terminal region, Blue bar: the conserved C-terminal domain, and ΔTPD1: TPD1 without the putative signal peptide. The 2.7-kb *TPD1* promoter was used for all constructs. (**B**-**G**) Compared with wild-type plants (**B**), primary inflorescences from 80% (96/120) of *TPD1*:*TPD1sp-*Δ*TPD1*/*tpd1* (**D**), 72% (36/50) of *TPD1*:*CLV3sp-*Δ*TPD1*/*tpd1* (**F**), and 70% (28/40) of *TPD1*:*PAP1sp-*Δ*TPD1*/*tpd1* (**G**) plants showed normal siliques, whereas 100% (88/88) of *TPD1*:Δ*TPD1/tpd1* plants (**E**) exhibited short siliques comparable to those of *tpd1* plants (**C**). Scale bars, 5 mm. (**H**-**M**) Pollen viability tests performed using Alexander pollen staining show functional pollen grains (red-stained) in wild-type (**H**), *TPD1*:*TPD1sp-*Δ*TPD1*/*tpd1* (**I**), *TPD1*:*CLV3sp-*Δ*TPD1/tpd1* (**L**), and *TPD1*:*PAP1sp-*Δ*TPD1*/*tpd1* (**M**) anthers, but not in *tpd1* (**J**) or *TPD1*:Δ*TPD1/tpd1* (**K**) anthers. Scale bars, 50 μm. (**N**-**S**) Semi-thin sections of stage-5 anthers showing normal anther cell differentiation in wild-type (**N**), *TPD1*:*TPD1sp-*Δ*TPD1*/*tpd1* (**O**), *TPD1*:*CLV3sp-*Δ*TPD1/tpd1* (**R**), and *TPD1*:*PAP1sp-*Δ*TPD1*/*tpd1* (**S**) anthers, but not in *TPD1*:*△TPD1*/*tpd1* (**Q**) or *tpd1* (**P**) anthers, which lacked tapetal cells and exhibited excess microsporocytes. E, epidermis; En, endothecium; ML, middle layer; T, tapetal cell; and M, microsporocyte. Scale bars, 10 μm.

If TPD1 acts as a ligand of EMS1, it should be secreted to the extracellular space and bind to the EMS1 LRR region. To test this, we first analyzed the effect of the putative signal peptide of TPD1 on its function in anther cell differentiation. Using the *TPD1* promoter, we created constructs in which the putative signal peptide of TPD1 was replaced with the known signal peptides, CLV3 and PAP1 [38,39] (Fig 4A). The resulting constructs were tested for complementation effects on fertility, pollen viability, and anther cell differentiation in the *tpd1* mutant background. Compared with wild-type plants (Fig 4B), the *tpd1* mutant was completely sterile (Fig 4C). Eighty percent (96/120) of *TPD1*:*TPD1sp-ΔTPD1*/*tpd1* plants exhibited normal fertility (Fig 4D), but no *TPD1*:*ΔTPD1*/*tpd1* plant (0/88) showed restoration of fertility (Fig 4E). Wild-type and *TPD1*:*TPD1sp-ΔTPD1*/*tpd1* anthers produced viable pollen grains (Fig 4H and 4I), whereas, similar to *tpd1* plants (Fig 4J), *TPD1*:*ΔTPD1/tpd1* plants did not produce any pollen (Fig 4K). Stage-5 anthers of wild-type (Fig 4N) and *TPD1*:*TPD1sp-ΔTPD1*/*tpd1* plants had normal microsporocytes surrounded by tapetum (Fig 1O), whereas *tpd1* (Fig 1P) and *TPD1*:*ΔTPD1/tpd1* anthers exhibited a total lack of tapetum but had excess microsporocytes (Fig 1Q).

Seventy two percent (36/50) of *TPD1*:*CLV3sp-*Δ*TPD/tpd1* and 70% (28/40) of *TPD1*:*PAP1sp-*Δ*TPD1*/*tpd1* plants exhibited rescued fertility (Fig 4F and 4G), produced viable pollen grains (Fig 4L and 4M), and formed normal anthers (Fig 4R and 4S). Together, our results suggest that the putative signal peptide of TPD1 and the N-terminal signal peptide-directed secretion of TPD1 are required for its function in anther cell fate determination.

### TPD1 is a secreted protein, and it is detected at the plasma membrane in the presence of EMS1

**Fig 5:**
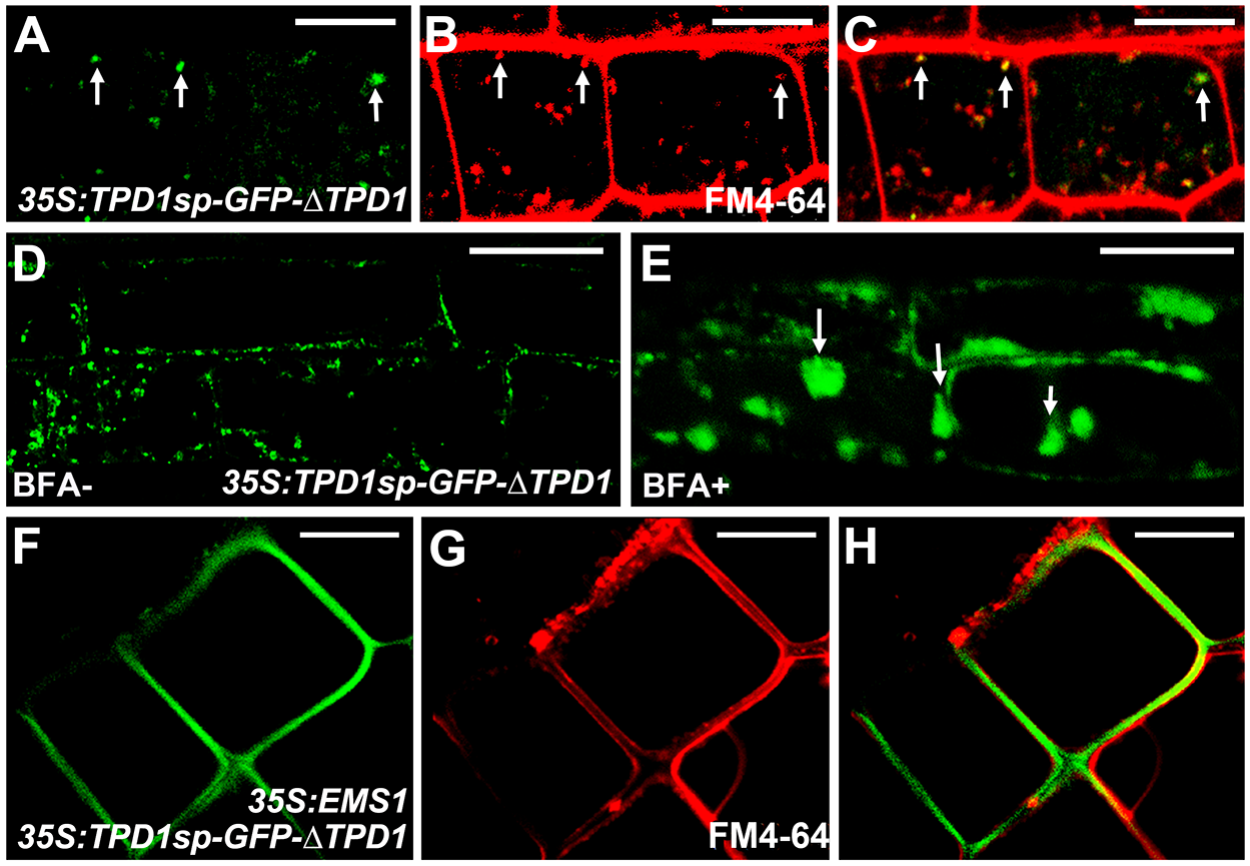
TPD1 is a secreted protein whose localization at the plasma membrane depends on EMS1. *35S*:*TPD1sp-GFP-ΔTPD1* and *35S*:*TPD1sp-GFP-ΔTPD1 35S*:*EMS1* transgenic plants were used to analyze the localization and secretion of TPD1 in root cells. (**A**-**C**) Confocal images of *35S*:*TPD1sp-GFP-ΔTPD1* root cells showing TPD1 proteins [arrows, (**A**)], FM4-64-stained trafficking vesicles [arrows, (**B**)], and GFP signals overlapping with trafficking vesicles [arrows, (**C**)]. (**D**, **E**) Confocal images of *35S*:*TPD1sp-GFP-ΔTPD1* root cells treated without (**D**) or with (**E**) BFA, which blocks the formation of Golgi-derived vesicles. Arrows in (**E**) indicate aggregated TPD proteins. (**F**-**H**) Confocal images of *35S*:*TPD1sp-GFP-ΔTPD1 35S*:*EMS1* root cells showing TPD1sp-GFP-ΔTPD1 proteins at the plasma membrane (**F**: GFP alone, **G**: FM4-64-stained, and **H**: merged). Scale bars, 10 μm.

To test whether TPD1 is a secreted protein, we generated *35S*:*TPD1sp-GFP-ΔTPD1* transgenic plants and analyzed the subcellular localization of TPD1 in root cells. We observed TPD1 proteins in trafficking vesicles but not at the plasma membrane (Fig 5A–5C). Treatment with brefeldin A (BFA), which blocks the formation of Golgi-derived vesicles [40], caused aggregation of TPD1 proteins (Fig 5D and 5E), suggesting that the TPD1 protein is contained within trafficking vesicles. Since *EMS1* is not expressed in the root [10], we generated *35S*:*TPD1sp-GFP-*Δ*TPD1 35S*:*EMS1* double transgenic plants. In the presence of EMS1, we observed TPD1 at the plasma membrane of root cells (Fig 5F–5H).

We obtained similar results using the *Arabidopsis* leaf protoplast system. TPD1 was observed in vesicle-like compartments of protoplasts transfected with *35S*:*TPD1sp-GFP-ΔTPD1* alone, and at the plasma membrane in the presence of the full-length EMS1 and the EMS1 LRR domain, but not the EMS1 kinase domain (S4 Fig). TPD1 proteins lacking the signal peptide (GFP-ΔTPD1) or with site mutations in the K135R136 dibasic site (TPD1sp-GFP-ΔTPD1 ^K135G R136G^) failed to localize to the plasma membrane regardless of the presence of EMS1 (S4 Fig).

Together, these results suggest that TPD1 is a secreted protein that localizes to the plasma membrane in an EMS1-dependent fashion.

### The localization of TPD1 and EMS1 in anthers suggests that TPD1 is a secreted protein ligand

**Fig 6:**
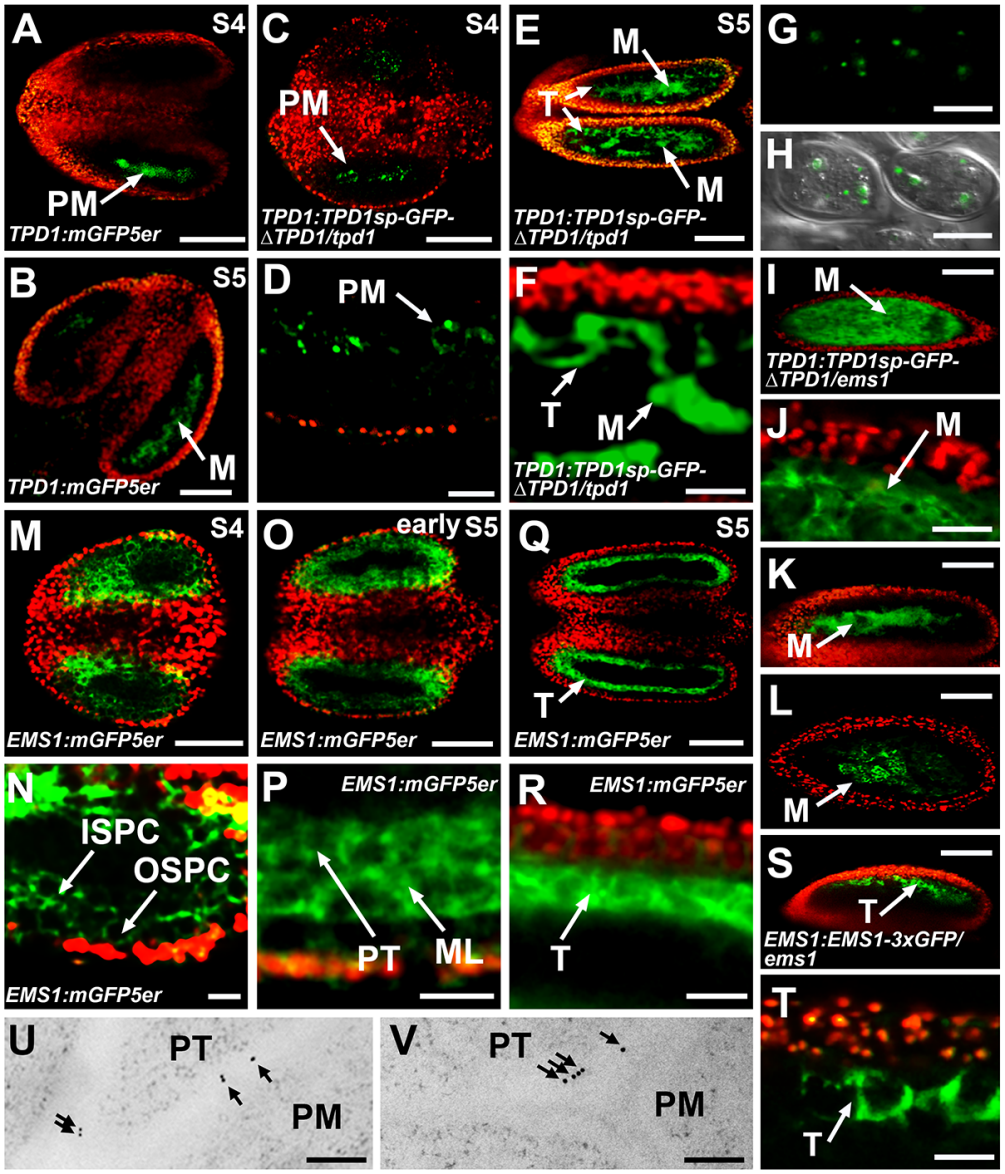
Localization of TPD1 and EMS1 in anthers. (**A**-**T**) Confocal images showing merges of red chlorophyll autofluorescence and green GFP signals, with the exceptions of (**G**) and (**H**). (**A**, **B**) In *TPD1*:*mGFP5er* stage-4 (**A**) and stage-5 (**B**) anthers, the *TPD1* promoter was active only in precursors of microsporocytes and microsporocytes, respectively. (**C**, **D**) In the *TPD1*:*TPD1sp-GFP-ΔTPD1/tpd1* stage-4 anther, TPD1 proteins were detected in precursors of microsporocytes (**D**, high magnification of **C**). (**E**, **F**) In the *TPD1*:*TPD1sp-GFP-ΔTPD1/tpd1* stage-5 anther, TPD proteins were mainly localized in microsporocytes, but were also detected at the surface of tapetal cells (**F**, high magnification of **E**). (**G**, **H**) TPD1 proteins were localized in vesicle-like compartments of microsporocytes isolated from *TPD1*:*TPD1sp-GFP-ΔTPD1/tpd1* stage-5 anthers (**H,** confocal image merged with DIC-viewed microsporocytes). (**I, J**) In the *TPD1*:*TPD1sp-GFP-ΔTPD1/ems1* stage-5 anther, the TPD1 localization domain was expanded and TPD1 proteins were evenly distributed in microsporocytes as these anthers lacked tapetal cells (**J**, high magnification of **I**). (**K, L**) In *TPD1*:*GFP-ΔTPD1* (**K**) and *TPD1*:*TPD1sp-GFP-ΔTPD1*^*K135G R136G*^ (**L**) stage-5 anthers, TPD1 proteins were restricted to microsporocytes, regardless of the presence of EMS1. (**M**, **N**) In the *EMS1*:*mGFP5er* stage-4 anther, the *EMS1* promoter was active in outer secondary parietal cells (OSPC) and inner secondary parietal cells (ISPC) (**N**, high magnification of **M**). (**O**, **P**) In the early *EMS1*:*mGFP5er* stage-5 anther, the *EMS1* promoter was active in the middle layer (ML) and precursors of tapetal cells (PT) (**P**, high magnification of **O**). (**Q**, **R**) In the *EMS1*:*mGFP5er* stage-5 anther, *EMS1* promoter activity was only detected in tapetal cells (T) (**R**, high magnification of **Q**). (**S**, **T**) In the *EMS1*:*EMS1-3xGFP/ems1* stage-5 anther, EMS1 proteins were only observed at surfaces of tapetal cells (**T** shows a higher magnification of **S**). (**U, V**) EM-immunolabeling results showing TPD1 (**U**) and EMS1 (**V**) proteins at the plasma membrane of precursors of tapetal cells from *TPD1*:*TPD1sp-GFP-*Δ*TPD1/tpd1* and *EMS1*:*EMS1-3xGFP/ems1* early stage-5 anthers, respectively. For each *GFP* fusion gene, at least 15 independent T2 plants were observed. Similar GFP signals were observed from >90% tested plants. The anther stage was determined by FM4-64 staining (See S5 Fig) after GFP images were acquired. ISPC, inner secondary parietal cell; M, microsporocyte; ML, middle layer; OSPC, outer secondary parietal cell; PM, precursor of microsporocyte; PT, precursor of tapetal cell; S, stage; and T, tapetal cell. (**A**-**C**, **E**, **I**, K, **L**, **M**, **O**, **Q, S**) Scale bars, 50 μm. (**D**, **F**, **J**, **N**, **P**, **R, T**) Scale bars, 20 μm. (**G**, **H**) Scale bars, 10 μm. (**U**, **V**) Scale bars, 0.2 μm.

Analysis of *TPD1*:*mGFP5er* [modified endoplasmic reticulum (ER)-localized GFP] plants showed that the *TPD1* promoter was active in precursors of microsporocytes (PM) at stage 4 (Fig 6A) and in microsporocytes (M) at stage 5 (Fig 6B). In *TPD1*:*TPD1sp-GFP-*Δ*TPD1/tpd1* plants, which showed normal anther development (Fig 1D and 1I), the TPD1 protein was present in PM at stage 4 (Fig 6C and 6D). At stage 5, TPD1 was observed not only in M but also at the surface of tapetal cells (T) (Fig 6E and 6F). Similar to our observations in root and leaf cells (Fig 5A–5C; S4 Fig), TPD1 was found in vesicle-like compartments of isolated M (Fig 6G and 6H). In the *ems1* mutant anther, the TPD1 localization domain was expanded, although TPD1 proteins were evenly observed only in microsporocytes because the *ems1* anther has excess microsporocytes but no tapetal cells (Fig 6I and 6J; S5 Fig). In *TPD1*:*GFP-ΔTPD1* and *TPD1*:*TPD1sp-GFP-ΔTPD1*^*K135G R136G*^ anthers, TPD1 proteins were restricted to M and were not found in T (Fig 6K and 6L), indicating that the secretion and cleavage of TPD1 are required for its localization in T. As described above, GFP-ΔTPD1 (lacking the signal peptide) and TPD1sp-GFP-ΔTPD1^K135G R136G^ (harboring site mutations in the K135R136 cleavage site) were also trapped in the cytoplasm of protoplasts, regardless of the presence of EMS1 (S4 Fig). Our findings suggest that TPD1 is synthesized in PM/M and secreted to their surrounding tapetal cells.

The *EMS1* promoter was active in both outer secondary parietal cells (OSPC) and inner secondary parietal cells (ISPC) at stage 4 (Fig 6M and 6N). Following the periclinal division of ISPC and their subsequent differentiation, the *EMS1* promoter was active in the middle layer (ML) and the precursors of tapetal cells (PT), which are derived from ISPC early in stage 5 (Fig 6O and 6P). Later in stage 5, *EMS1* promoter activity was restricted to T (Fig 6Q and 6R). The EMS1 protein was observed at the surface of T in stage-5 anthers (Fig 6S and 6T), suggesting that it is localized at the plasma membrane of these cells. After the GFP signals were analyzed, the anthers were stained with FM4-64 dye, and anther stages and cell types were determined from optical sections under confocal microscopy (S5 Fig).

To further examine the subcellular localizations of TPD1 and EMS1, we carried out EM-immunolabeling experiments. Our results showed that the TPD1 and EMS1 proteins were localized to the plasma membrane of PT in early stage-5 anthers (Fig 6U and 6V; S6 Fig).

Together, these results suggest that TPD1 is synthesized in PM/M, secreted from these cells to PT/T, and stabilized by interacting with EMS1 at the plasma membrane of PT/T. The activated TPD1-EMS1 signaling may initially promote parietal cell division and then determine the fate of functional tapetal cells.

### TPD1 activates the periclinal division of anther wall cells

**Fig 7:**
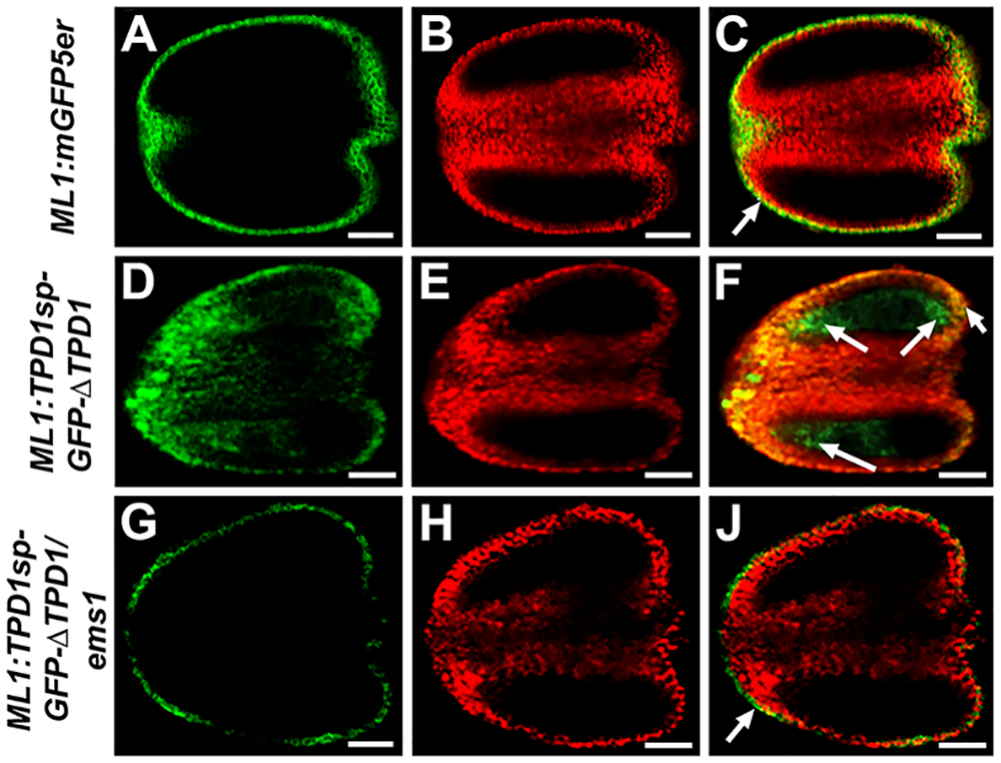
The movement of TPD1 from epidermis to inner cell layers in *ML1*:*TPD1sp-GFP-*Δ*TPD1* anthers. (**A, D, G**) show the GFP signal; (**B, E, H**) show chlorophyll autofluorescence; and (**C, F, J**) show merged images. (**A**-**C**) *ML1*:*mGFP5er* stage-5 anther showing GFP signals only in the epidermis (arrow). (**D**-**F**) *ML1*:*TPD1sp-GFP-*Δ*TPD1* stage-5 anther showing an expanded GFP signal domain (arrows) that suggests the TPD1 proteins have moved to subepidermal cells. (**G**-**I**) *ML1*:*TPD1sp-GFP-*Δ*TPD1/ems1* stage-5 anther showing GFP signals only in the epidermis (arrow). Twenty *ML1*:*mGFP5er*, 15 *ML1*:*TPD1sp-GFP-*Δ*TPD1*, and 19 *ML1*:*TPD1sp-GFP-*Δ*TPD1/ems1* T2 plants were analyzed. Similar GFP signals were observed in all examined plants of each group. Scale bars, 20 μm.

To test whether TPD1 acts as a secreted protein ligand to control anther cell division and tapetal cell fate determination, we manipulated the expression of *TPD1* using the *Arabidopsis MERISTEM LAYER1* (*ML1*) promoter, which is specifically active in the epidermis of various organs [41]. In *ML1*:*GFP* anthers, the *ML1* promoter was specifically active in anther epidermis (Fig 7A–7C). In *ML1*:*TPD1sp-GFP-*Δ*TPD1* anthers, in contrast, we observed GFP signals in both anther epidermis and subepidermal cell layers (Fig 7D–7F). In *ML1*:*TPD1sp-GFP-*Δ*TPD1*/*ems1* anthers, the GFP signal was only observed in anther epidermis (Fig 7G and 7H). Our data suggest that the TPD1 protein is capable of traveling through cell layers from epidermis to inner cells in the presence of EMS1.

**Fig 8:**
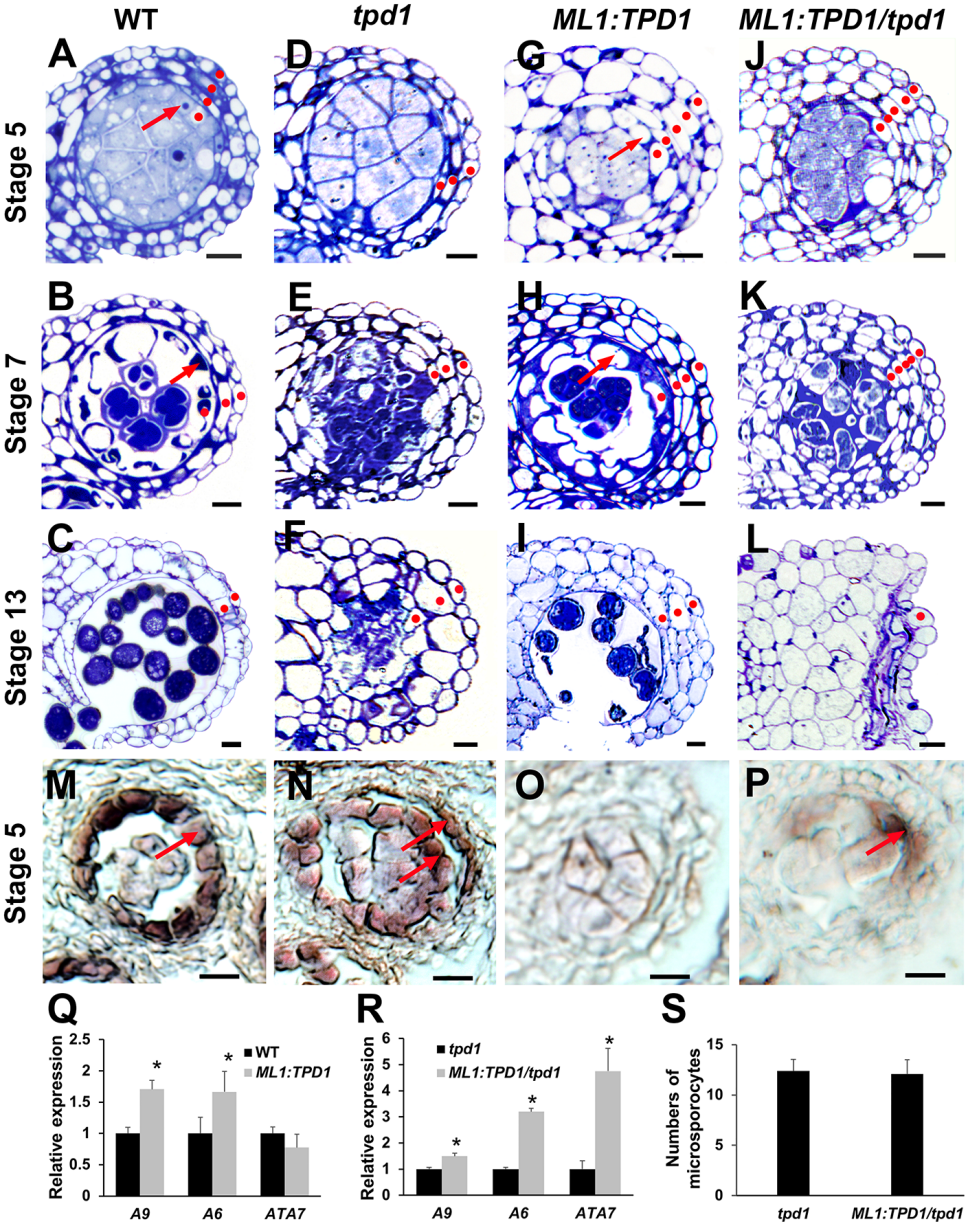
Ectopic expression of TPD1 in anther epidermis promotes periclinal anther wall cell division and tapetal cell differentiation. (**A-C**) Anther lobes of wild-type plants at stages 5 (**A**), 7 (**B**), and 13 (**C**). (**D-F**) Anther lobes of *tpd1* plants at stages 5 (**D**), 7 (**E**), and 13 (**F**). (**G-I**) Anther lobes of *ML1*:*TPD1* plants at stages 5 (**G**), 7 (**H**), and 13 (**I**). (**J-L**) Anther lobes of *ML1*:*TPD1/tpd1* plants at stages 5 (**J**), 7 (**K**), and 13 (**L**). Red dots indicate the somatic anther wall cell layers. Arrows in (**A**, **B**, **G**, **H**) indicate tapetal cells. (**M-P**) At stage 5, *in situ* hybridization shows expression of the tapetal cell marker gene, *A9*, in a single layer of tapetal cells of the wild-type anther (**M**, arrow), in two cell layers of the *ML1*:*TPD1* anther (**N**, arrows), and in a few cells of the *ML1*:*TPD1/tpd1* anther (**P**, arrow). No *A9* expression was found in the *tpd1* anther (**O**). Scale bars, 10 μm. (**Q, R**) QRT-PCR results showing expressions of the tapetal cell marker genes, *A9*, *A6*, and *ATA7*, in wild-type and *ML1*:*TPD1* anthers (**Q**), and in *tpd1* and *ML1*:*TPD1/tpd1* anthers (**R**). Stars indicate a significant difference (*P*<0.01). (**S**) Numbers of microsporocytes per transverse section taken at the mid-point of the abaxial lobe in *tpd1* (n = 20) and *ML1*:*TPD1/tpd1* (n = 20) anthers at stage 5. Thirty *ML1*:*TPD1* plants and 30 *ML1*:*TPD1/tpd1* plants were subjected to semi-thin sectioning. Eighty percent (24/30) of *ML1*:*TPD1* plants and 90% (27/30) of *ML1*:*TPD1/tpd1* plants showed similar phenotypes in anther development.

Wild-type anthers had four somatic cell layers at stage 5 (Fig 8A). Three cell layers were visible at stage 7, reflecting degeneration of the middle layer (Fig 8B). Two cell layers remained at stage 13, reflecting degeneration of the tapetal cells (Fig 8C). In contrast, *tpd1* anthers contained just three somatic cell layers (the epidermis, endothecium, and middle layer) throughout their development (Fig 8D–8F). Ectopic expression of *TPD1* (*ML1*:*TPD1*) caused production of more than four somatic cell layers in both wild-type (Fig 8G and 8H) and *tpd1* (Fig 8J and 8K) plants. Interestingly, at stage 13, three cell layers were observed in *ML1*:*TPD1* anthers (Fig 8I), whereas all subepidermal anther wall cells were dead at this point in *ML1*:*TPD1/tpd1* anthers (Fig 8L).

Previous *in situ* hybridization results showed that the ectopic expression of *TPD1* triggered expression of the tapetal cell marker gene, *A9* [20], in both the tapetum and its neighboring cells in the wild-type background (Fig 8M and 8N). *A9* was not expressed in the *tpd1* anther (Fig 8O), while in the *ML1*:*TPD1/tpd1* anther, it was expressed in a few cells adjacent to microsporocytes (Fig 8P). Our qRT-PCR results further confirmed that the expression of *TPD1* in epidermis significantly increased the expression of the tapetal cell marker genes, *A9*, *A6*, and *ATA7* (Fig 8Q and 8R), suggesting that TPD1 promotes tapetal cell differentiation in the presence of EMS1. The number of microsporocytes in *ML1*:*TPD1/tpd1* anthers was similar to that in *tpd1* anthers (Fig 8S), indicating that the increase in somatic cell layers did not suppress microsporocyte proliferation in the absence of the tapetum.

Taken together, our results suggest that TPD1 first promotes the periclinal division of anther wall cells and subsequently determines tapetal cell fate in the presence of EMS1.

### Microsporocyte-derived TPD1 mediates the acquisition of tapetal cell fate

**Fig 9:**
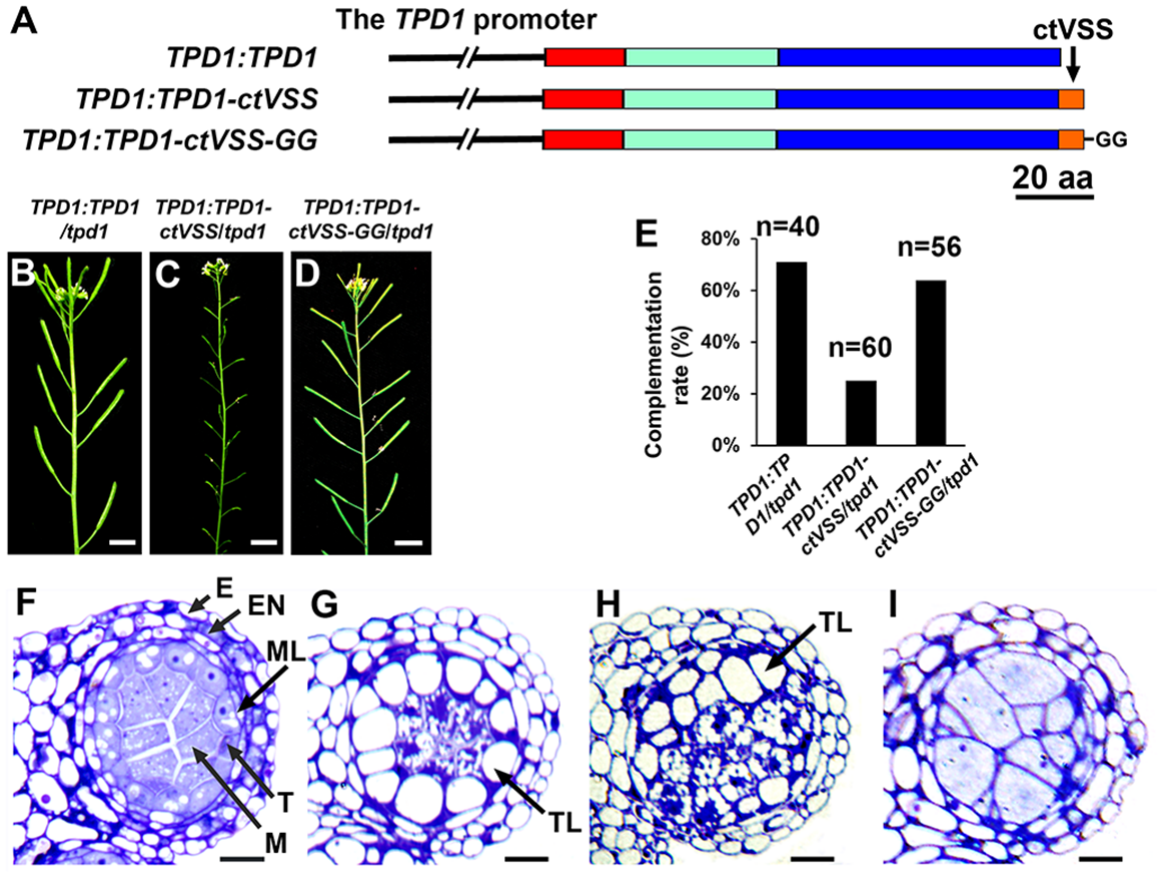
Missorting of TDP1 demonstrates that microsporocyte-derived TPD1 mediates the acquisition of tapetal cell fate. (**A**) Schematic diagrams showing the structures of the *TPD1*:*TPD1*, *TPD1*:*TPD1-ctVSS*, and *TPD1*:*TPD1-ctVSS-GG* constructs. Red bar: the TPD1 putative signal peptide, Cyan bar: the non-conserved N-terminal region, Blue bar: the conserved C-terminal domain, Orange bar, the C-terminal vacuole sorting signal (ctVSS), and “GG”: two glycines added to ctVSS. (**B-D**) Primary inflorescences showing normal fertilities (indicated by long siliques) were obtained from *TPD1*:*TPD1/tpd1* (**B**) and *TPD1*:*TPD1-ctVSS-GG*/*tpd1* (**D**) plants, whereas *TPD1*:*TPD1-ctVSS*/*tpd1* plants were sterile (as indicated by short siliques) (**C**). Scale bars, 1 cm. (**E**) Complementation rates (%) of *TPD1*:*TPD1/tpd1* (n = 40), *TPD1*:*TPD1-ctVSS/tpd1* (n = 60), and *TPD1*:*TPD1-ctVSS-GG/tpd1* (n = 56) plants. (**F**) A stage-5 anther lobe from a *TPD1*:*TPD1/tpd1* plant showing normal anther cell differentiation. (**G**-**I**) Stage-5 anther lobes from *TPD1*:*TPD1-ctVSS*/*tpd1* plants showing defective anther cell differentiation: a monolayer of vacuolated tapetal-like cells and degenerating microsporocytes (**G**), delaminated vacuolated tapetal-like cells and degenerating microsporocytes (**H**), and a lack of tapetal cells coupled with the presence of excess microsporocytes, which is similar to the *tpd1* phenotype (**I**). Thirty sterile *TPD1*:*TPD1-ctVSS*/*tpd1* plants were subjected to semi-thin sectioning. Among them, most (21/30) exhibited the anther phenotype shown in (**G**), five exhibited that shown in (**H**), and four exhibited that shown in (**I**). E, epidermis; En, endothecium; M, microsporocyte; ML, middle layer; T, tapetal cell; and TL, tapetal-like cells. Scale bars, 10 μm.

To test whether TPD1 mediates communication between microsporocytes and tapetal cells, we generated a protein in which TPD1 was fused with the ctVSS C-terminal vacuolar sorting signal (Fig 9A), which directs proteins into vacuoles [39]. The addition of two glycines to the ctVSS sequence disables this sorting function (Fig 9A) [39]. Seventy five percent (30/40) of *TPD1*:*TPD1*/*tpd1* (Fig 9B and 9E) and 68% (38/56) of *TPD1*:*TPD1-ctVSS-GG*/*tpd1* (Fig 9D and 9E) plants exhibited normal fertility, whereas 75% (45/60) of *TPD1*:*TPD1-ctVSS*/*tpd1* plants were sterile (Fig 9C and 9E). Although viable pollen was observed in *TPD1*:*TPD1*/*tpd1* (S7 Fig) and *TPD1*:*TPD1-ctVSS-GG*/*tpd1* anthers, no pollen was found in the *TPD1*:*TPD1-ctVSS*/*tpd1* anther (S7 Fig). Further analyses showed that *TPD1*:*TPD1-ctVSS*/*tpd1* anthers exhibited various phenotypes, such as degenerated microsporocytes surrounded by a monolayer of vacuolated tapetal-like cells (Fig 9F and 9G), degenerated microsporocytes adjacent to delaminated vacuolated tapetal-like cells (Fig 9H), or a lack of recognizable tapetal cells but the presence of excess microsporocytes (Fig 9I). Moreover, our qRT-PCR results showed that the expression levels of *TPD1* were similar in *TPD1*:*TPD1*/*tpd*, *TPD1*:*TPD1-ctVSS*/*tpd1*, and *TPD1*:*TPD1-ctVSS-GG*/*tpd1* anthers (S7 Fig), supporting the idea that the observed anther defects were caused by abnormal sorting of TPD1 proteins.

From these results, we suggest that the TPD1-mediated communication between precursors of microsporocytes/microsporocytes and early anther wall cells is required for tapetal cell differentiation and the maintenance of cell integrity.

### Interdependence of tapetal cell and microsporocyte differentiation

**Fig 10:**
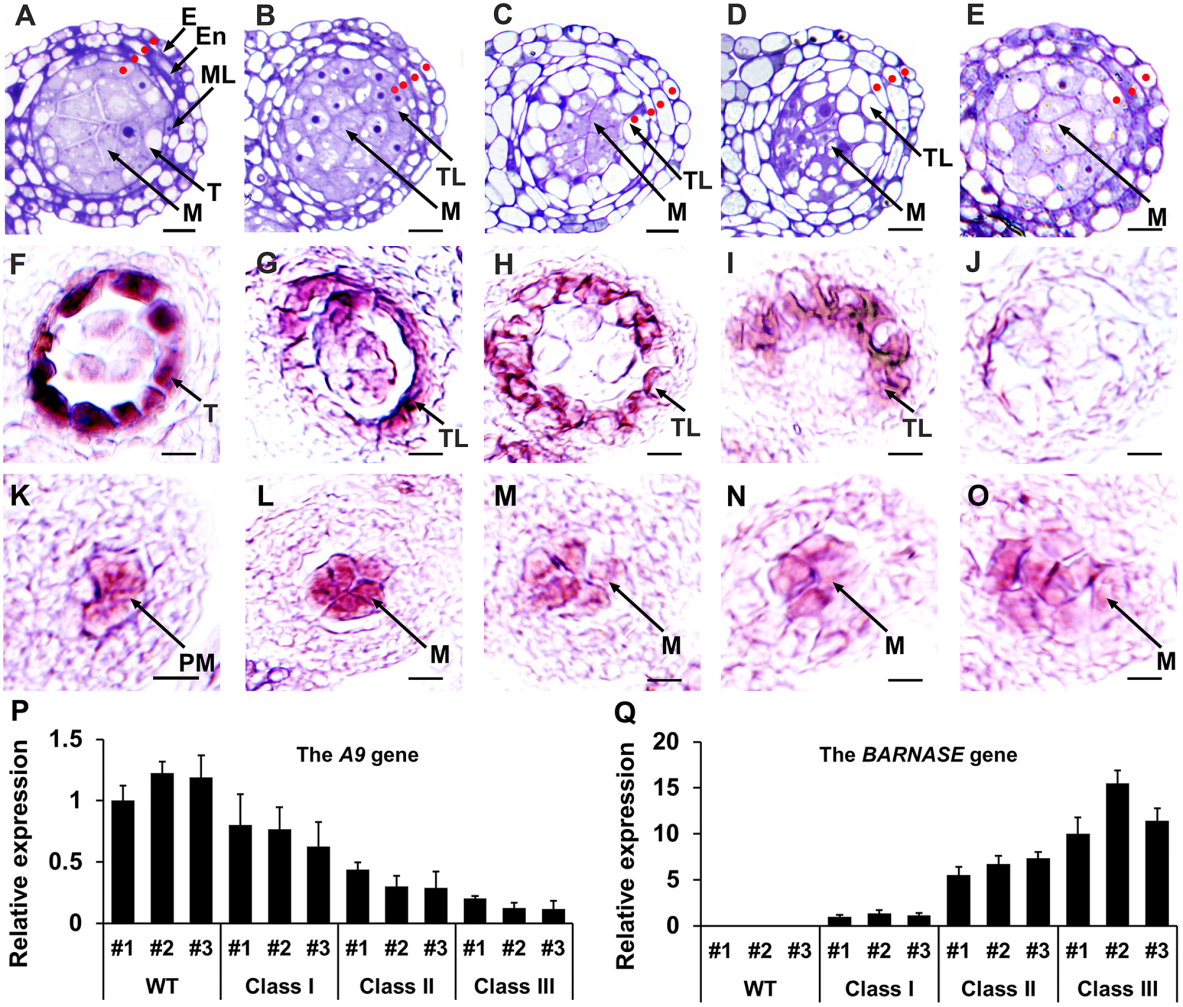
Genetic ablation of microsporocytes shows the interdependence of tapetal cell and microsporocyte differentiation. (**A**) A wild-type anther lobe at stage 5 showing four layers of anther wall cells (indicated by red dots, the same hereinafter) and microsporocytes. (**B-E**) *SDS*:*SDS-BANASE* anther lobes at stage 5, which we divided into three classes. Class I: four somatic cell layers, including one organized single-cell layer that surrounds the microsporocytes and is made of cells that are morphologically similar to tapetal cells (**B**). Class II: four somatic cell layers, including a monolayer of vacuolated tapetal-like cells (**C**) and three somatic cell layers that contains delaminated vacuolated tapetal-like cells (**D**). Degenerating microsporocytes are observed in Class-II anthers. Class III: three somatic cell layers and excess microsporocytes (**E**). Among 60 T1 plants analyzed by semi-thin section, 16.7% (10/60) were Class I, 70.0% (42/60) were Class II, and 13.3% (8/60) were Class III. (**F**-**J**) *In situ* hybridization results showing that the expression of the tapetal cell marker gene, *A9* at stage 5 was strong in the tapetum of the wild-type anther (**F**), but was progressively decreased in tapetal-like cells from Class-I (**G**) and Class-II (**H**, **I**) *SDS*:*SDS-BANASE* anthers. No *A9* expression was detected in the Class-III *SDS*:*SDS-BANASE* anther (**J**). (**K**-**O**) *In situ* hybridization results showing the expression of the microsporocyte marker gene, *SDS*, in anthers. In wild-type anthers, *SDS* expression was weak at stage 4 in precursors of microsporocytes (**K**), but strong at stage 5 in microsporocytes (**L**). *SDS* was weakly expressed in the microsporocytes of *SDS*:*SDS-BANASE* anthers (**M-O**). The *SDS* expression domain was relatively expanded in *SDS*:*SDS-BANASE* Class-II and -III anthers (**N, O**). E, epidermis; En, endothecium; M, microsporocyte; ML, middle layer; PM, precursor of microsporocyte; T, tapetal cell; and TL, tapetal-like cell. Scale bars, 10 μm. (**P**, **Q**) We used qRT-PCR to examine expression levels of *A9* and *BARNASE* in anthers from three representative transgenic plants of each class. From Class I to Class III anthers, the expression of *A9* progressively decreased (**P**), while that of *BARNASE* increased (**Q**).

Although the lack of tapetal cells ultimately causes failure of pollen formation, it does not reportedly affect microsporocyte differentiation in the *tpd1*, *mil2*, *mac1*, *ems1*, *msp1*, *serk1 serk2*, or *bam1 bam2* mutants [10,12,13,16,42–44], suggesting that microsporocyte specification is independent of anther wall development. The *SOLO DANCERS* (*SDS*) gene is specifically expressed in microsporocytes [45, 46]. To test how reproductive cells (e.g. precursors of microsporocytes/microsporocytes) affect somatic wall cell differentiation in anthers, we generated *SDS*:*SDS-BARNASE* plants, in which microsporocyte differentiation was specifically impaired by the expression of the toxic gene, *BARNASE*, in these cells [45,46]. All *SDS*:*SDS-BARNASE* plants were male-sterile due to a lack of pollen [46]. Based on semi-thin sectioning of 60 *SDS*:*SDS-BARNASE* T1 plants, however, we were able to divide the plants into three classes based on their anther phenotypes at stage 5. In class I plants (16.7%, 10/60), stage-5 anthers harbored four somatic cell layers that resembled those of the wild-type anther (Fig 10A and 10B). In class II plants (70.0%; 42/60), stage-5 anthers harbored four somatic cell layers, including either a monolayer of vacuolated tapetal-like cells (Fig 10C) or three somatic cell layers with delaminated vacuolated tapetal-like cells (Fig 10D). In addition, degenerating microsporocytes were observed (Fig 10C and 10D). In class III plants (13.3%; 8/60), stage-5 anthers lacked tapetal cells but exhibited the presence of excess microsporocyte-like cells; in this, they resembled *ems1* and *tpd1* mutant anthers, with the addition of early microsporocyte degeneration (Fig 10E).

We further performed *in situ* hybridization to examine the expressions of the *A9* and *SDS* genes. Compared with the strong expression of the tapetum marker gene, *A*9, in the tapetum of the wild-type stage-5 anther (Fig 10F), *A9* expression was progressively decreased in *SDS*:*SDS-BARNASE* Class-I to Class-II anthers (Fig 10G–10I) at stage 5, and no *A9* expression was detected in the Class-III anther (Fig 10J). Our qRT-PCR results agreed with the *in situ* hybridization results (Fig 10P).

In wild-type anthers, *SDS* expression was weak in precursors of microsporocytes at stage 4 (Fig 10K) but strong in microsporocytes at stage 5 (Fig 10L). Stage-5 *SDS*:*SDS-BARNASE* anthers exhibited decreased *SDS* expression relative to wild-type anthers (Fig 10M–10O). The expression domain of *SDS* was similar in wild-type and Class-I anthers (Fig 10M), but relatively expanded in Class-II (Fig 10N) and Class-III (Fig 10O) anthers. Our qRT-PCR results showed that the severity of tapetal cell defects corresponded to the expression level of *BARNASE* (Fig 10Q).

Our results indicate that a monolayer of tapetal cells cannot be fully differentiated when microsporocytes are affected by SDS-BARNASE. Weak SDS-BARNASE function does not affect ISPC division but still leads to abnormal differentiation of tapetal cells. In the presence of moderate SDS-BARNASE function, there are two possibilities: if ISPC division occurs normally, a monolayer of vacuolated tapetal-like cells is formed; if ISPC division is not complete, however, delaminated vacuolated tapetal-like cells are produced. Finally, strong and early SDS-BARNASE function bars ISPC division, resulting in a lack of tapetal-like/tapetal cells but the presence of excess microsporocytes.

In summary, our results suggest that the formation of a monolayer of PT and the complete acquisition of tapetal cell fate depend on normal microsporocyte development, while tapetal cells in turn suppress microsporocyte proliferation.

## Discussion

In this report, we provide several lines of strong evidence supporting our contention that TPD1 is a small secreted cysteine-rich protein ligand for the EMS1 LRR-RLK, thereby expanding the repertoire of this receptor class. Cysteine-rich peptides/proteins, such as EPF1, EPF2 [31], EPFL9 [32,33,47], LAT52 [35], STIG1 [34], S-locus cysteine rich protein (SCR)/*S*-locus protein 11 (SP11) [48], RALF [49], LURE1, LURE2 [50], and EC1 [51], serve as ligands for a large group of receptor kinases. The LAT52, LURE1, LURE2, and EC1 peptides remain intact [35,50,51], whereas RALF and STIG1 are cleaved [34,49]. In *Arabidopsis*, 10 out of 34 AtRALFs contain RR dibasic sites (RRXL). Mutations in these dibasic sites impair the cleavage of AtRALF1 and AtRALF23 [36,52], the latter of which is known to be cleaved at the dibasic site by the AtS1P protease (subtilase) [52]. A 7-kD C-terminal STIG1 peptide, possibly arising from cleavage at the dibasic site, was identified by electrospray ionization–mass spectrometry in tomato [34]. Our present results add to this knowledge by showing that the TPD1 C terminus, which contains the dibasic sites, might be important for the stability or translation of this small protein ligand. *TPD1*:*TPD1sp-GFP-ΔTPD1* functioned as well as the native *TPD1* gene. In contrast, *TPD1*:*TPD1sp-ΔTPD1 -GFP* did not complement the *tpd1* phenotype; it was transcribed (S8 Fig), but no TPD1-GFP protein was detected. Similar results were also obtained from the *TPD1*:*TPD1-GUS* gene (S9 Fig), suggesting that the addition of a large protein to the TPD1 C terminus may abolish its function, possibly via protein synthesis failure or misfolding-induced degradation. In planta, the MAC1 protein is an 18-kD intact protein that does not seem to be processed beyond removal of the putative signal peptide [15]. Our results suggest that TPD1 differs from MAC1 in that it is cleaved at the C terminal K135R136 site, resulting in a 13-kD small protein ligand. Our data showed that TPD1 was not cleaved in the absence of the signal peptide or when the K135R136 cleavage site is mutated. In addition, the uncleaved TPD1 was not localized at the plasma membrane or in trafficking-like vesicles even in the presence of EMS1, suggesting that TPD1 cannot enter the secretion secretory pathway unless it has undergone normal processing.

LRR is a 20-30-residue motif that has been identified in thousands of proteins across all organisms [53]. Recent crystal structure studies on plant LRRs have demonstrated the molecular mechanisms underlying the activation of LRR-RLK complexes by brassinolide (BL), which is the most active brassinosteroid ligand, and by the 22-residue oligopeptide ligand, flg22 [54–57]. The binding of BL to the BRI1 island domain induces the formation of an interface that subsequently activates the SERK1-BRI1 (co-receptor and receptor) complex. flg22 binds to the concave surface of the FLS2 LRR domain across 14 LRRs and is sandwiched between FLS2 and BAK1 (SERK3) LRR ectodomains. The gluing effect of flg22 causes full activation of the FLS2-BAK1 complex. Our present and previous [9] studies identified two TPD1 binding sites in the EMS1 LRR ectodomain. Here, we hypothesize that the binding of TPD1 to the EMS1 LRR2-constituted domain results in a conformational rearrangement of the interface between EMS1 and its unknown co-receptor, which then becomes more competent for additional binding of TPD1, leading to the full activation of EMS1 signaling. As TPD1 represents a novel small protein ligand for LRR-RLKs, future studies on the structures and dynamic interactions of TPD1 and EMS1 could support the development of a new paradigm for the small-protein-ligand-induced activation of LRR-RLK signaling complexes.

**Fig 11:**
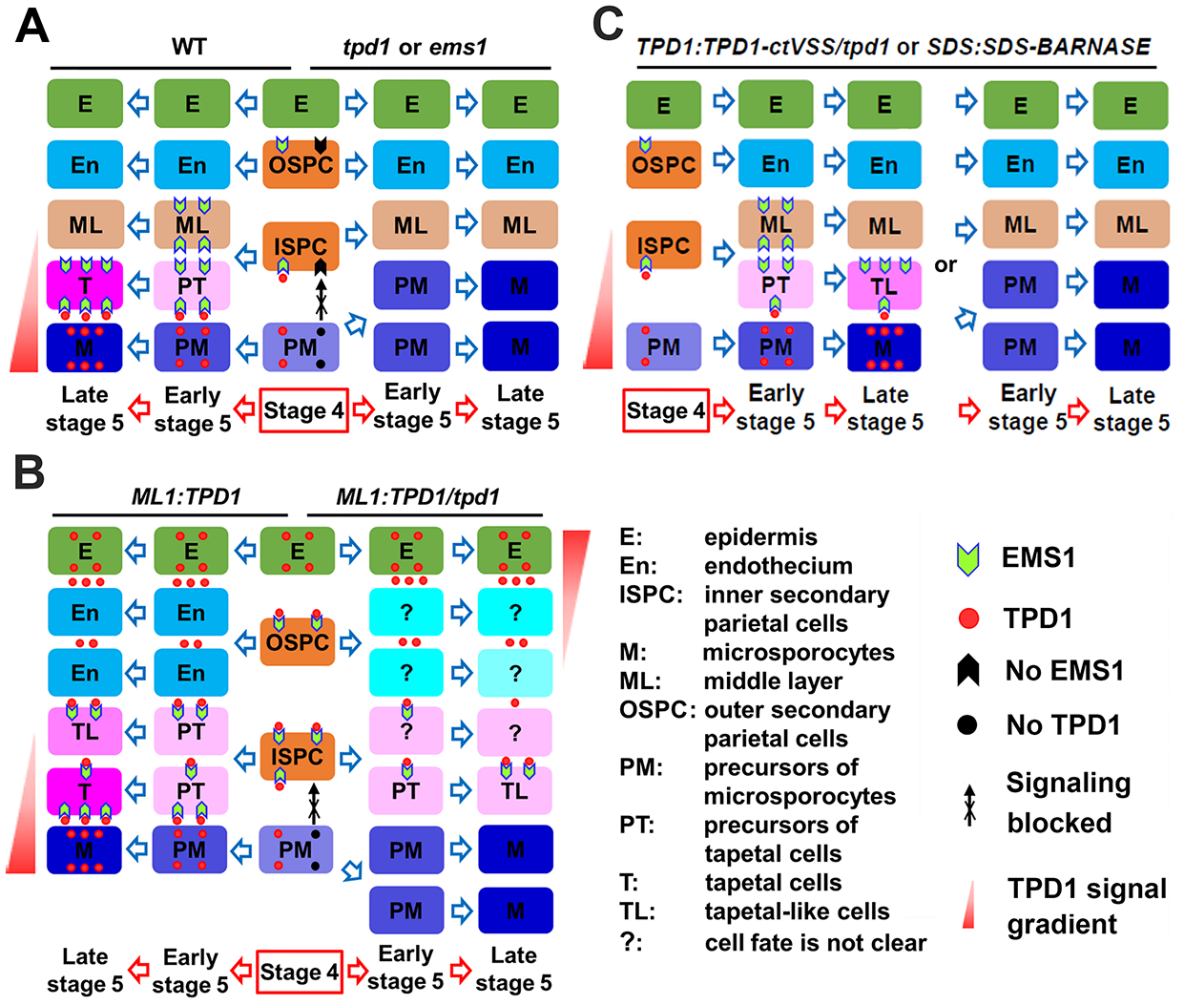
Working model of TPD1-EMS1 signaling in anther development. (**A**) Early anther development in wild-type, *tpd1*, and *ems1* plants. In wild-type stage-4 anthers, EMS1 is expressed in outer and inner secondary parietal cells (OSPC and ISPC), whereas TPD1 is synthesized in precursors of microsporocytes (PM). The binding of secreted TPD1 to EMS1 at the plasma membrane of ISPC promotes the periclinal division of these cells, generating the middle layer (ML) and precursors of tapetal cells (PT). Early in stage 5, PT perceive sufficient TPD1, which sustains TPD1-EMS1 signaling and leads to the differentiation of functional tapetal cells (T) late in stage 5. Differentiated T in turn suppress the proliferation of microsporocytes (M). In the *tpd1* or *ems1* mutant, TPD1-EMS1 signaling is blocked. The failure of ISPC division leads to an absence of T, which results in the formation of excess M. (**B**) Early anther development in *ML1*:*TPD1* and *ML1*:*TPD1/tpd1* plants. TPD1 is highly expressed in epidermis (E) and moves to regions beneath OSPC and ISPC. Activated TPD-EMS1 signaling promotes the periclinal division of OSPC and ISPC. In *ML1*:*TPD1* anthers (wild-type background), OSPC generate two layers of endothecium (En). ISPC and their daughter cells receive sufficient TPD1-EMS1 signaling, which leads to the formation of extra T/tapetal-like cells (TL). In *ML1*:*TPD1/tpd1* anthers (*tpd1* background), no TPD1 is synthesized in PM. Low-level TPD1-EMS1 signaling can promote OSPC and ISPC division, but only the few PT that receive sufficient TPD1-EMS1 signaling differentiate into TL. Without T, extra anther wall cell layers present but fail to suppress M proliferation in *ML1*:*TPD1/tpd1* anthers. (**C**) Early anther development in *TPD1*:*TPD1-ctVSS/tpd1* and *SDS*:*SDS-BARNASE* plants. In most *TPD1*:*TPD1-ctVSS/tpd1* anthers, there is either sufficient TPD1 to promote ISPC division but PT cannot differentiate into functional T, or there is insufficient TPD1 to promote ISPC division, resulting in a complete lack of T and the presence of excess M. In most *SDS*:*SDS-BARNASE* anthers, arrest of M differentiation occurs late and ISPC still divide to form ML and PT, but the differentiation of functional T is not completed due to the lack of TPD1. In a few *SDS*:*SDS-BARNASE* anthers, in contrast, M differentiation is arrested early, ISPC division fails, and excess M are produced. Question marks indicate instances where the cell fate is not clear.

The tapetum, which is essential for pollen development, consists of a monolayer of polynucleate cells [58–60] that comes to surround successive stages of microsporocytes, tetrads, microspores, and developing pollen grains as anther development progresses [1,2,5,8]. In *Arabidopsis*, a monolayer of precursors of tapetal cells (PT) is established early in stage 5, and the differentiation of functional tapetal cells is completed late in stage 5 [7,10]. Our present results show that the earliest expression of EMS1 is observed in outer and inner secondary parietal cell layers (OSPC and ISPC) at stage 4, when TPD1 also becomes detectable in precursors of microsporocytes (PM) (Fig 11A). We propose that the presence of sufficient TPD1-EMS1 in ISPC initially promotes their periclinal division to generate two cell layers, the middle layer (ML) and PT. Thereafter, sustained TPD1-EMS1 signaling in PT directs the complete differentiation of functional tapetal cells at late stage 5 (Fig 11A).

In *ems1* and *tpd1* anthers, the loss of TPD1 or EMS1 leads to failure of ISPC division, with the result that such anthers exhibit no tapetal cells but an excess of microsporocytes (Fig 11A). Our model is further supported by the observation that manipulation of TPD1 expression and secretion affects the formation of anther wall cell layers and the fate determination of tapetal cells. Epidermis-expressed TPD1 can travel to regions beneath OSPC and ISPC, promoting the periclinal division of these cells and eventually triggering the formation of extra anther wall layers (Fig 11B). Interestingly, in the *tpd1* mutant background, epidermis-expressed TPD1 is sufficient to stimulate periclinal division of OSPC and ISPC but insufficient to complete the differentiation of functional tapetal cells. This may explain why epidermis-expressed TPD1 in *tpd1* plants was found to increase the expression of tapetal cell marker genes, but such plants experience precocious death of such cells (Fig 8L and 8R). In contrast, the inhibition of TPD1 by the engineered missorting of TPD1 or the ablation of microsporocytes was associated with incomplete tapetal cell differentiation or a lack of tapetal cells (Fig 11C). A monolayer of vacuolated tapetal cells was often observed in *TPD1*:*TPD1-ctVSS/tpd1* and *SDS*:*SDS-BARNASE* anthers, suggesting that PT formed but failed to differentiate into functional tapetal cells in the absence of sufficient TPD1. When most TPD1 expression was blocked, PT failed to form and no tapetal cells were observed. Our results collectively support the idea that TPD1-EMS1 signaling is initially required to promote the periclinal division of ISPC to form PT, and then subsequently to determine the final tapetal cell fate.

A previous report found that the expression of *EMS1* under the control of the tapetum-specific *A9* promoter could rescue the tapetum in *ems1* anthers, suggesting that TPD1-EMS1 stimulates the proliferation of a small population of tapetal founder cells (or primary tapetal cells) to form a monolayer of tapetal cells via anticlinal cell division [20]. This did not support the idea that TPD1-EMS1 determines tapetal cell fate. If tapetal founder cells exist, wild-type and *ems1* anthers should have a similar number of tapetal founder cells. The previous study, however, found that each *ems1* anther lobe contained an average of three tapetal founder cells, while wild-type anthers contained a greater number of tapetal founder cells that were organized as a monolayer [20]. Although tapetal founder cells possess some features of differentiated tapetal cells, they are precociously degenerated, indicating that tapetal founder cells are not fully differentiated tapetal cells. On the other hand, anatomically, a monolayer of tapetal cells is hardly established by a small number of tapetal founder cells in one location, since plant cells do not move. The *A9* promoter becomes active in the tapetum at stage 5 when *EMS1* is expressed. Here, the high-level *A9*-promoter-driven expression of *EMS1* could be sufficient to promote the periclinal division of quiescent ISPC and the final differentiation of PT into functional tapetal cells. In some circumstances, ISPC might be directly differentiated into tapetal cells without the periclinal division step. Together, these results support the notion that TPD1-EMS1 is required for tapetal cell fate determination.

Various studies have proposed different interpretations regarding the control of microsporocyte proliferation. In maize, MAC1 represses the excessive proliferation of archesporial cells (AR), either by binding an unknown receptor kinase in AR or by indirectly inducing neighboring somatic cells to produce a repressor of AR proliferation [15,18]. Our results show that EMS1 is not present in AR or microsporocyte/microsporocyte precursors, which rules out the possibility that TPD1-EMS1 signaling directly represses microsporocyte proliferation. If TPD1 activates a different receptor complex in microsporocytes, these cells would not overproliferate in *ems1* anthers. If the involved receptor complexes share components, however, then the lack of EMS1 could impair receptors in both somatic and germinal cells. In *Arabidopsis*, disruption of the *RPK2 LRR*-*RLK* gene results in the absence of the middle layer but does not affect the number of microsporocytes [61]. Conversely, loss-of-function mutants of the *EMS1*, *SERK1*/*2*, and *BAM1*/*2 LRR*-*RLK* genes produce anthers that possess excess microsporocytes but lack either the tapetum or all anther wall cell layers except the epidermis [42,43,62]. Our present results show that although *ML1*:*TPD1 tpd1* anthers produce extra somatic cell layers, they still form excess microsporocytes in the absence of the tapetum monolayer (Figs 8J, 8K, 8S and 11B). Moreover, when TPD1 expression is largely blocked via missorting of TPD1 or ablation of microsporocytes, the anthers do not produce tapetum, but instead form excess microsporocytes (Figs 9I, 10E and 11C). Taken together, these results show that the tapetum is required to limit the proliferation of microsporocytes but not other cells of the anther wall.

Monocots and dicots exhibit conservation of regulators, but may differ in their sexual reproduction strategies. For example, disruption of *SPOROCYTELESS* (*SPL*)**/***NOZZLE* (*NZZ*), which encodes a novel transcription repressor, blocks the differentiation of primary sporogenous cells, resulting in an absence of microsporocytes in *Arabidopsis* [63,64]. SPL/NZZ orthologs exist in monocots, such as maize and rice, but their functions in sexual reproduction have not yet been identified [65]. Loss of function in the ROXY1 and ROXY2 glutaredoxins causes sporogenous cell differentiation to fail in the two adaxial anther lobes of *Arabidopsis* [66]. In contrast, the MSCA1 glutaredoxin acts earlier in maize, as shown by the observation that the archesporial cells of *msca1* anthers differentiate into vasculature instead of sporogenous cells [18]. In maize, MAC1 activates the periclinal division of PPC to form endothecium and SPC. In *Arabidopsis*, however, our present results show that TPD1 is not required for PPC division, but rather functions later to promote the periclinal division of ISPC to form ML and the tapetum. Loss-of-function mutants in the *MSP1*, *MIL2*, and *MAC1* genes exhibit an anther phenotype similar to those of *tpd1* and *ems1* plants and additionally produce extra megaspore mother cells (MMC) in their ovules, suggesting that MSP1, MIL2, and MAC1 suppress the proliferations of both microsporocytes and MMC in monocots [13,14,17]. Although neither *tpd1* nor *ems1* plants exhibited developmental defects in their ovules [10,12], our recent report showed that ectopic *ML1*-promoter-driven expression of *TPD1* affects ovule cell division by regulating auxin signaling and cell-cycle-related genes [67]. It is worth noting that in the anthers of monocots such as maize, the cells of the anther wall (e.g., PPC, SPC, and PT) are established as monolayers at or near the time they are first formed, whereas the corresponding cells in *Arabidopsis* are not observed as monolayers until tapetal cell differentiation is almost complete [1,2,5,7,8,10,18]. Cytologically, reproductive AR and sporogenous cells are more recognizable in monocot anthers than in *Arabidopsis* anthers. Overall, the differences in anther development between monocots and dicots might be associated with functional differences in the identified regulators.

Seed plants have evolved a sophisticated anther wall structure to ensure the success of sexual reproduction. In animals, signals from somatic cells influence the fate determination of reproductive cells, and vice versa [68]. In plant anthers, somatic cells of the anther wall are not required for the differentiation of reproductive microsporocytes [4,10–12,42,43,61,62], but signals from reproductive cells do appear to determine the complexity of anther wall cells. LRR-RLK-linked signals, including TPD1-EMS1, are essential for the communication of reproductive and somatic cells during anther development [4,9–11,13,42,43,61]. In the future, additional studies on LRR-RLK-linked signaling should provide new insights into the molecular mechanisms underlying somatic and reproductive cell fate determination in plants.

## Materials and Methods

### Plant materials and growth conditions

*Arabidopsis thaliana* [Landsberg *erecta* (L*er*)] plants were grown in Metro-Mix 360 soil (Sun-Gro Horticulture City State) in growth chambers under a 16-hour light/8-hour dark photoperiod, at 22°C and 50% humidity.

### Generation of constructs and transgenic plants

For genetic analyses and protein localization studies, the *TPD1*, *EMS1*, and *ML1* promoters [10,12,67] were individually introduced into the pENTR/D-TOPO vector (Invitrogen), and the corresponding genes were cloned and inserted. Overlapping PCR was used to replace the TPD1 signal peptide with the CLV3 or PAP1 signal peptide [38,39], and to generate site mutations. To generate the TPD1-GFP fusion protein, GFP was inserted right after the TPD1 signal peptide to generate TPD1-GFP-ΔTPD1. All constructs in pENTR/D-TOPO vectors were sub-cloned into Gateway Binary vectors (pGBWs or pEARLEYs) using the Gateway LR recombinase II enzyme mix (Invitrogen). The resulting constructs were then transformed into *Agrobacterium* strain GV3101. Transformations were performed using the floral dip method [69]. The transformants were screened on 50 μg/mL kanamycin and 25 μg/mL hygromycin or 1% Basta (PlantMedia).

pSAT vectors [70] were used to generate constructs for subcellular localization and BiFC assays in the protoplast system. To generate the BiFC constructs, nEYFP (the N-terminal part of EYFP) was fused to full-length TPD1, mature TPD1, or mutated TPD1 at the cleavage site following the TPD1 signal peptide (Fig 3A). Similarly, cEYFP (the C-terminal part of EYFP) was fused to full-length EMS1 and a series of mutants that were truncated after the EMS1 signal peptide (Fig 3E). Each construct contained the EMS1 signal peptide and transmembrane domain. Phusion High-fidelity DNA polymerase (New England Biolabs) was used for all PCRs. Detailed information for all constructs and the primers used to generate them is presented in S1 and S2 Tables.

### Pollen staining and anther semi-thin sections

Alexander pollen staining and anther semi-thin sectioning were conducted as described previously [9,10].

### Protoplast transfection

Transient expression of proteins in *Arabidopsis* protoplasts was performed as described previously [71]. The *35S*:*EYFP* plasmid was used to monitor transfection efficiencies. At least three replicates were performed for each assay.

### Western blotting, RNA *in situ* hybridization, and qRT-PCR

Young buds were collected from wild-type plants, transgenic plants, and transfected protoplasts, proteins were prepared, and Western blotting was performed as described previously [9], utilizing rabbit anti-GFP (Torrey Pines Biolabs) as the primary antibody and anti-rabbit IgG-HRP (Cell Signaling Technology) as the secondary antibody.

RNA *in situ* hybridization was performed as previously described [10,72] using anthers from wild-type, *tpd1* mutant, and transgenic plants. The SP6/T7 DIG RNA Labeling Kit (Roche) was used to generate sense and antisense probes against *A9* and *SDS*.

RNA was extracted from young buds of wide-type, *tpd1* mutant, and transgenic plants using an RNeasy Plant Mini Kit (Qiagen). RNA quantification, reverse transcription, real-time PCR (DNA Engine Opticon 2 system), and data analysis were performed as described previously [72] to examine the expression levels of the anther-specific genes, *A9*, *A6*, and *ATA7*, and the *BARNASE* gene (for primers, see S2 Table). The constitutively expressed *ACTIN2* gene was examined as a control. Three independent experiments were performed.

### EM-immunogold-labeling

The EM-immunogold-labeling was performed as described previously [73]. Young anthers from *TPD1*:*TPD1sp-GFP-*Δ*TPD1/tpd1* and *EMS1*:*EMS1-3xGFP/ems1* transgenic plants were dissected and processed by the high-pressure freezing procedure. Rabbit anti-GFP (Torrey Pines Biolabs) was used as the primary antibody, while anti-rabbit IgG conjugated to 15-nm gold particles (Ted Pella) was used as the secondary antibody. Controls were run in parallel without the primary antibody.

### Microscopy

Images of anther semi-thin sections were visualized and photographed using an Olympus BX51 microscope equipped with an Olympus DP 70 digital camera. For confocal microscopy, samples were observed with a Leica TCS SP2 laser scanning confocal microscope using a 63×/1.4 water immersion objective lens. A 488-nm laser was used to excite GFP, EYFP, chlorophyll, and FM4-64. Emission were captured using PMTs set at 505–530 nm, 500–550 nm, and 660–760, 644–719 nm, respectively. For visualization of cell membranes and trafficking vesicles, roots were treated with 20 μM FM4-64 (Invitrogen) or 30 μM BFA (Sigma-Aldrich) for 5–30 min, washed, and then observed. For localization of TPD1 and EMS1, anthers were dissected from young buds and mounted in water. For immunogold-labeling, images were obtained with an FEI CM 120 electron microscope.

## Supporting Information

S1 Table Constructs generated in this study. (PDF)

S2 Table Primers used in this study. (PDF)

S1 Fig Alignment of TPD1 and its orthologs in other species. ClustalW2 was used for sequence alignment. Sequences were retrieved from the NCBI database. Numbers and frames indicate the positions of cysteine residues. The domain definition is based on the Arabidopsis TPD1 sequence. Red line: the putative signal peptide, Cyan line: the non-conserved N-terminal region, Blue line: the conserved C-terminal domain. Arrowhead indicates the putative dibasic cleavage site.
(TIF)

S2 Fig Analyses of TPD1 processing in the leaf protoplast transient system. (**A**) Schematic diagrams showing the structures of the *TPD1sp-GFP-ΔTPD1*, *GFP-ΔTPD1*, and *TPD1sp-GFP-ΔTPD1*^*K135G R136G*^ constructs. Red bar: the TPD1 putative signal peptide (Sp), Green bar: GFP, Cyan bar: the non-conserved N-terminal region, Blue bar: the conserved C-terminal domain, ΔTPD1: TPD1 without the putative signal peptide, and Pink line: K135GR135G mutations. (**B**) Western blotting was used to examine the processing of GFP-fused TPD1 proteins extracted from transfected *35S*:*EMS1* leaf protoplasts. *35S*:*EMS1* shows no band; *35S*:*GFP-ΔTPD1 35S*:*EMS1* exhibits a 45-kD band; *35S*:*TPD1sp-GFP-ΔTPD1*^*K135G R136G*^ *35S*:*EMS1* exhibits a 48-kD band; and *35S*:*TPD1sp-GFP-ΔTPD1 35S*:*EMS1* exhibits a 41-kD band (arrow).
(TIF)

S3 Fig The function of TPD1-EMS1 signaling depends on the interaction of TPD1 with EMS1 in the first three LRRs. To examine the importance of the interaction of TPD1 with EMS1 in the first three LRRs, *35S*:*EMS1*, *35S*:*TPD1*, *35S*:*TPD1 35S*:*EMS1*^*K104N*^, and *35S*:*TPD1 35S*:*EMS1* plants were generated and analyzed. The *35S*:*EMS1* plant appeared similar to the wild-type plant. The *35S*:*TPD1* plant produced short and wide siliques, but was nearly normal in stature. The *35S*:*TPD1 35S*:*EMS1* plant was dwarf and had twisted leaves, stem, inflorescences, and siliques. In contrast, the *35S*:*TPD1 35S*:*EMS1*^*K104N*^ plant resembled the *35S*:*TPD1* plant.
(TIF)

S4 Fig Analyses of the localization and secretion of TPD1 in leaf protoplasts. (**A**) Schematic diagrams showing the truncated and mutated versions of TPD1 and EMS1. For TPD1 constructs, Red bar: the TPD1 putative signal peptide (Sp), Green bar: GFP, Cyan bar: the non-conserved N-terminal region, Blue bar: the conserved C-terminal domain, ΔTPD1: TPD1 without the putative signal peptide, and Pink line: K135GR135G mutations. For EMS1 constructs, Red bar: the EMS1 putative signal peptide (Sp), Dodger blue bar: leucine-rich repeat (LRR), Brown bar: the transmembrane domain (TM), Olive green bar: kinase domain (KD), and Yellow bar: EYFP. (**B**, **C**) Confocal images showing TPD1sp-GFP-ΔTPD1 inside the leaf protoplast [**B**, GFP signal; **C**, GFP merged with chlorophyll autofluorescence (red)]. Arrows indicate GFP signals in trafficking vesicle-like compartments. (**D**-**I**) Merged confocal images. (**D**) Full-length EMS1-EYFP at the plasma membrane. (**E**, **F**) TPD1sp-GFP-ΔTPD1 at the plasma membrane in the presence of full-length EMS1 (**E**) and the EMS1 LRR domain (**F**). (**G**) TPD1sp-GFP-ΔTPD1 is not observed at the plasma membrane in the presence of the EMS1 kinase domain (KD). (**H, I**) GFP-ΔTPD1 (**H**) and TPD1sp-GFP-ΔTPD1^K135G R136G^ (**I**) were not found at the plasma membrane or in trafficking vesicle-like compartments, regardless of the presence of EMS1. Scale bars, 10 μm.
(TIF)

S5 Fig Determination of anther stages by optical sectioning and confocal microscopy. For staging, anthers were dissected in 20 μM FM4-64 solution and stained for 1 hour. (**A**) Confocal image showing epidermis (E), outer secondary parietal cells (OSPC), inner secondary parietal cells (ISPC), and precursors of microsporocytes (PM) in a stage-4 wild-type anther. (**B**) Confocal image showing E, endothecium (En), the middle layer (ML), precursors of tapetal cells (PT), and PM in the early stage-5 wild-type anther. (**C**) Confocal image showing E, En, ML, tapetal cells (T), and microsporocytes (M) in the stage-5 wild-type anther. The green line encloses microsporocytes. (**D**) Confocal image showing normal-looking E, En, ML, but a large domain of M in the *ems1* stage-5 anther. No T was observed in the *ems1* stage-5 anther. The green line encloses microsporocytes. Scale bars, 50 μm.
(TIF)

S6 Fig Determination of the anther stage and the intensity of gold particles following EM-immunolabeling. **(A, B)** Semi-thin sections showing the early-stage-5 *TPD1*:*TPD1sp-GFP-*Δ*TPD1/tpd1* (A) and *EMS1*:*EMS1-3xGFP/ems1* (**B**) anthers used for EM-immunolabeling (Fig 6T and 6U). Scale bars, 10 μm. (**C**) Statistics for the numbers of gold particles per micron at the plasma membrane and between cells. The length of the plasma membrane was measured using the ImageJ software. Stars indicate that the numbers of gold particles in *TPD1*:*TPD1sp-GFP-*Δ*TPD1/tpd1* and *EMS1*:*EMS1-3xGFP/ems1* anthers are significantly higher than that in wild-type anthers (*P*<0.01).
(TIF)

S7 Fig TPD1-ctVSS impairs pollen viability, and the expression levels of the *TPD1* transgene are similar among examined plants. (**A**-**C**) Pollen viability was tested by Alexander pollen staining: (**A**) The *TPD1*:*TPD1*/*tpd1* anther exhibits functional pollen grains, (**B**) no pollen is observed in the *TPD1*:*TPD1-ctVSS*/*tpd1* anther, and (**C**) normal pollen grains are seen in the *TPD1*:*TPD1-ctVSS-GG*/*tpd1* anther. Scale bars, 50 μm. (**D**) qRT-PCR was used to examine *TPD1* expression in anthers of six plants each from the *TPD1*:*TPD1*/*tpd1* complemented lines, *TPD1*:*TPD1-ctVSS*/*tpd1* sterile lines, and *TPD1*:*TPD1-ctVSS-GG*/*tpd1* complemented lines. The results showed that the expression levels of *TPD1* were similar in all tested plants.
(TIF)

S8 Fig Analysis of *TPD1sp-ΔTPD1-GFP* expression. RT-PCR was used to examine the expression of the *TPD1sp-ΔTPD1-GFP* fusion gene in anthers. Four lines of *TPD1*:*TPD1sp-ΔTPD1-GFP/tpd1* plants were tested. *TPD1sp-ΔTPD1-GFP* expression was detected in all four transgenic lines but not in wild-type (WT) plants. The *ACTIN2* gene was used as the internal standard.
(TIF)

S9 Fig Analysis of TPD1sp-ΔTPD-GUS expression. GUS staining was used to examine the expression of TPD1sp-ΔTPD-GUS in anthers. Twenty *TPD1*:*TPD1sp-ΔTPD-GUS* plants were analyzed, but none showed any positive GUS signal. (**A**) A stage-5 anther showing no GUS signal. (B) A transverse section of a stage-5 anther showing no GUS signal.
(TIF)
