# Supplementary material for: Control of Anther Cell Differentiation by the Small Protein Ligand TPD1 and Its Receptor EMS1 in Arabidopsis
Source: PLoS Genet. 2016 Aug 18;12(8):e1006147. doi: 10.1371/journal.pgen.1006147 (PMC4990239; doi:10.1371/journal.pgen.1006147)
Supplement: S1 Table — (PDF) [file pgen.1006147.s001.pdf]

Supplementary Table 1: Constructs generated in this study

Constructs	Cloning method	Template	Primers	Cloning sites	Construct backbone
pENTR-TPD1:	TOPO cloning	F6I7 (TPD1 BAC)	zp199+zp200		pENTR/D-TOPO
pENTR-TPD1:TPD1	Ligation	U13545 (TPD1 cDNA)	zp541+zp396	KpnI+XbaI	pENTR-TPD1:
TPD1:TPD1	LR recombination	pENTR-TPD1:TPD1			pGWB1
pENTR-TPD1:ΔTPD1	Ligation	U13545 (TPD1 cDNA)	zp397+zp396	KpnI+XbaI	pENTR-TPD1:
TPD1:ΔTPD1	LR recombination	pENTR-TPD1:ΔTPD1			pGWB1
pENTR-TPD1:CLV3sp-ΔTPD	Ligation	U13545 (TPD1 cDNA)	zp415+zp396	KpnI+XbaI	pENTR-TPD1:
TPD1:CLV3sp-ΔTPD	LR recombination	pENTR-TPD1:CLV3sp-ΔTPD			pGWB1
pENTR-TPD1:PAP1sp-ΔTPD1		T22O13 (PAP1 BAC)<br>U13545 (TPD1 cDNA)	zp393+zp394<br>zp395+zp396	overlapping PCR	
	Ligation	(zp393+zp394)+(zp395+zp396)	zp393+zp396	KpnI+XbaI	pENTR-TPD1:
TPD1:PAP1sp-ΔTPD1	LR recombination	pENTR-TPD1:PAP1sp-ΔTPD1			pGWB1
pENTR-TPD1:TPD1-ctVSS	Ligation	U13545 (TPD1 cDNA)	zp541+zp389	KpnI+XbaI	pENTR-TPD1:
TPD1:TPD1-ctVSS	LR recombination	pENTR-TPD1:TPD1-ctVSS			pGWB1
pENTR-TPD1:TPD1-ctVSS-GG	Ligation	U13545 (TPD1 cDNA)	zp541+zp390	KpnI+XbaI	pENTR-TPD1:
TPD1:TPD1-ctVSS-GG	LR recombination	pENTR-TPD1:TPD1-ctVSS-GG			pGWB1
pENTR-TPD1:ΔC1	Ligation	U13545 (TPD1 cDNA)	zp541+zp1829	KpnI+XbaI	pENTR-TPD1:
ΔC1	LR recombination	pENTR-TPD1:ΔC1			pGWB1
pENTR-TPD1:ΔC2	Ligation	U13545 (TPD1 cDNA)	zp541+zp1987	KpnI+XbaI	pENTR-TPD1:
ΔC2	LR recombination	pENTR-TPD1:ΔC2			pGWB1
pENTR-TPD1:ΔC3	Ligation	U13545 (TPD1 cDNA)	zp541+zp1988	KpnI+XbaI	pENTR-TPD1:

ΔC3	LR recombination				pGWB1
pENTR-TPD1:ΔN1	Ligation	U13545 (TPD1 cDNA)<br>U13545 (TPD1 cDNA)<br>(zp541+zp1931)+(zp1932+zp396)	zp541+zp1931<br>zp1932+zp396<br>zp541+zp396	overlapping PCR<br>KpnI+XbaI	pENTR-TPD1:
ΔN1	LR recombination	pENTR-TPD1:ΔNC1			pGWB1
pENTR-TPD1:ΔN2	Ligation	U13545 (TPD1 cDNA)<br>U13545 (TPD1 cDNA)<br>(zp541+zp1933)+(zp1934+zp396)	zp541+zp1933<br>zp1934+zp396<br>zp541+zp396	overlapping PCR<br>KpnI+XbaI	pENTR-TPD1:
ΔN2	LR recombination	pENTR-TPD1:ΔNC2			pGWB1
pENTR-TPD1:C77S	Ligation	U13545 (TPD1 cDNA)<br>U13545 (TPD1 cDNA)<br>(zp541+zp2077)+(zp2076+zp396)	zp541+zp2077<br>zp2076+zp396<br>zp541+zp396	overlapping PCR<br>KpnI+XbaI	pENTR-TPD1:
C77S	LR recombination	pENTR-TPD1:C77S			pGWB1
pENTR-TPD1:C107S	Ligation	U13545 (TPD1 cDNA)<br>U13545 (TPD1 cDNA)<br>(zp541+zp2079)+(zp2078+zp396)	zp541+zp2079<br>zp2078+zp396<br>zp541+zp396	overlapping PCR<br>KpnI+XbaI	pENTR-TPD1:
C107S	LR recombination	pENTR-TPD1:C107S			pGWB1
pENTR-TPD1:C111S	Ligation	U13545 (TPD1 cDNA)<br>U13545 (TPD1 cDNA)<br>(zp541+zp2081)+(zp2080+zp396)	zp541+zp2081<br>zp2080+zp396<br>zp541+zp396	overlapping PCR<br>KpnI+XbaI	pENTR-TPD1:
C111S	LR recombination	pENTR-TPD1:C111S			pGWB1
pENTR-TPD1:C120S	Ligation	U13545 (TPD1 cDNA)<br>U13545 (TPD1 cDNA)<br>(zp541+zp2007)+(zp2006+zp396)	zp541+zp2007<br>zp2006+zp396<br>zp541+zp396	overlapping PCR<br>KpnI+XbaI	pENTR-TPD1:
C120S	LR recombination	pENTR-TPD1:C120S			pGWB1
pENTR-TPD1:C142S	Ligation	U13545 (TPD1 cDNA)<br>U13545 (TPD1 cDNA)<br>(zp541+zp2083)+(zp2082+zp396)	zp541+zp2083<br>zp2082+zp396<br>zp541+zp396	overlapping PCR<br>KpnI+XbaI	pENTR-TPD1:

C142S	LR recombination	pENTR-TPD1:C142S			pGWB1
pENTR-TPD1:C175S	Ligation	U13545 (TPD1 cDNA)	zp541+zp1918	KpnI+XbaI	pENTR-TPD1:
C175S		pENTR-TPD1:C175S			
35S:TPD1sp-GFP	Ligation	35S:TPD1	zp977+zp978	NgoMIV+AgeI	pEGAD
35S:TPD1sp-GFP-ΔTPD1	Ligation	U13545 (TPD1 cDNA)	zp570+zp571	EcoRI+SmaI	35S:TPD1sp-GFP
35S:GFP-ΔTPD1	Ligation	U13545 (TPD1 cDNA)	zp570+zp571	EcoRI+SmaI	pEGAD
TPD1:TPD1sp-ΔTPD1	Ligation	U13545 (TPD1 cDNA)	zp541+zp47	KpnI+XbaI	pENTR-TPD1:
TPD1:TPD1sp-ΔTPD1-GFP	LR recombination	TPD1:TPD1sp-ΔTPD1			pGWB4
TPD1:TPD1sp-ΔTPD1-GUS	LR recombination	TPD1:TPD1sp-ΔTPD1			pGWB3
pENTR-TPD1:TPD1sp-GFP-ΔTPD1	Ligation	35S:TPD1sp-GFP-ΔTPD1	zp541+zp396	KpnI+XbaI	pENTR-TPD1:
pENTR-TPD1:GFP-ΔTPD1	Ligation	35S:GFP-ΔTPD1	zp1029+zp396	KpnI+XbaI	pENTR-TPD1:
pENTR-TPD1:TPD1sp-GFP-$\Delta TPD1^{K135GR136G}$		35S:TPD1sp-GFP-ΔTPD1	zp541+zp2094	overlapping PCR	
		35S:TPD1sp-GFP-ΔTPD1	zp2093+zp396		
	Ligation	(zp541+zp2094)+(zp2093+zp396)	zp541+zp396	KpnI+XbaI	pENTR-TPD1:
TPD1:TPD1sp-GFP-[illegible]	LR recombination	pENTR-TTPD1:TPD1sp-GFP-[illegible]			pGWB1
pENTR-ΔN1	TOPO cloning	U13545 (TPD1 cDNA)	zp2433+zp396		pENTR/D-TOPO
pENTR-ΔC3	TOPO cloning	U13545 (TPD1 cDNA)	zp569+zp2432		pENTR/D-TOPO
pENTR-TPD1:TPD1sp-GFP-ΔN1	Ligation	pENTR-TPD1:TPD1sp-GFP-ΔTPD1+pENTR-ΔN1		EcoRI+AscI	pENTR/D-TOPO
pENTR-TPD1:TPD1sp-GFP-ΔC3	Ligation	pENTR-TPD1:TPD1sp-GFP-ΔTPD1+pENTR-ΔC3		EcoRI+AscI	pENTR/D-TOPO
TPD1:TPD1sp-GFP-ΔN1	LR recombination	pENTR-TPD1:TPD1sp-GFP-ΔN1			pGWB1
TPD1:TPD1sp-GFP-ΔC3	LR recombination	pENTR-TPD1:TPD1sp-GFP-ΔC3			pGWB1
35S:TPD1sp-GFP-ΔTPD1	Ligation	35S:TPD1sp-GFP-ΔTPD1	zp1036+zp396	KpnI+XbaI	pSAT6-mRFP-N1

35S:GFP-ΔTPD1	Ligation	35S:TPD1sp-GFP-ΔTPD1	zp1029+zp396	KpnI+XbaI	pSAT6-mRFP-N1 (pE3025)
35S:TPD1sp-GFP-ΔTPD1^{K135GR136G}	Ligation	pENTR-TPD1:TPD1sp-GFP-ΔTPD1^{K135GR136G}	zp1036+zp396	KpnI+XbaI	pSAT6-mRFP-N1
EMS1-EYFP	Ligation	T28J14 (EMS1 BAC)	zp802+zp804	Xho I + Kpn I	pSAT6-EYFP-N1 (pE3225)
pENTR-EMS1	TOPO cloning	T28J14 (EMS1 BAC)	zp52+zp73		pENTR/D-TOPO
35S:EMS1	LR Recombin ation	pENTR-EMS1			pGWB2
EMS1sp-cEYFP		T28J14 (EMS1 BAC)<br>pSAT1-cEYFP-C1-B	zp1070+zp1066<br>zp1065+zp1064	overlapping PCR	
	Ligation	(zp1070+zp1066)+(zp1 065+zp1064)	zp1070+zp1064	NcoI+XhoI	pSAT1-cEYFP-C1-B
cEYFP-EMS1	Ligation	T28J14 (EMS1 BAC)	zp803+zp2120	XhoI+KpnI	EMS1sp-cEYFP
cEYFP-LRR	Ligation	T28J14 (EMS1 BAC)	zp803+zp1041	XhoI+KpnI	EMS1sp-cEYFP
cEYFP-KD	Ligation	T28J14 (EMS1 BAC)	zp2119+zp2120	XhoI+KpnI	EMS1sp-cEYFP
cEYFP-LRR-I	Ligation	T28J14 (EMS1 BAC)	zp803+zp1080	XhoI+KpnI	EMS1sp-cEYFP
cEYFP-ΔLRR-I	Ligation	T28J14 (EMS1 BAC)	zp1071+zp1072	SacII+BamH I	EMS1sp-cEYFP-LRR-I
cEYFP-ΔLRR-II	Ligation	T28J14 (EMS1 BAC)	zp1073+zp1041	XhoI+KpnI	EMS1sp-cEYFP
cEYFP-LRR-III		T28J14 (EMS1 BAC)	zp803+zp1165	XhoI+KpnI	EMS1sp-cEYFP
cEYFP-ΔLRR-III		T28J14 (EMS1 BAC)	zp1071+zp1072	SacII+BamH I	EMS1sp-cEYFP-LRR-III
cEYFP-LRR-IV	Ligation	T28J14 (EMS1 BAC)	zp1073+zp1074	XhoI+KpnI	EMS1sp-cEYFP
cEYFP-ΔLRR-IV	Ligation	T28J14 (EMS1 BAC)	zp1071+zp1072	SacII+BamH I	EMS1sp-cEYFP-LRR-IV
cEYFP-ΔLRR-V	Ligation	T28J14 (EMS1 BAC)	zp1075+zp1041	XhoI+KpnI	EMS1sp-cEYFP
pENTR-EMS1^{K104N}		T28J14 (EMS1 BAC)<br>T28J14 (EMS1 BAC)	zp979+zp1164<br>zp1163+zp272	overlapping PCR	
	TOPO cloning	(zp979+zp1164)+(zp11 63+zp272)	zp979+zp272		pENTR/D-TOPO
35S:EMS1^{K104N}	LR recombination	pENTR-EMS1(K104N)			pGWB2
cEYFP-EMS1^{K104N}	Ligation	pENTR-EMS1(K104N)	zp803+zp1080	XhoI+KpnI	ΔLRR-II-cEYFP
cEYFP-BRI1-LRR	Ligation	F23K16 (BRI1 BAC)	zp1076+zp1077	XhoI+KpnI	EMS1sp-cEYFP
pENTR-TPD1sp-GFP-ΔTPD1	TOPO cloning	TPD1sp-GFP-ΔTPD1	zp541+zp396		pENTR/D-TOPO

pENTR-TPD1sp-nEYFP-ΔTPD1	Ligation	TPD1sp-GFP-ΔTPD1	zp1082+zp1084	AgeI+EcoRI	pENTR-TPD1sp-GFP-
nEYFP-TPD1	Ligation	pENTR-TPD1sp-nEYFP-ΔTPD1	zp541+zp396	KpnI+XbaI	pSAT6-mRFP-N1
nEYFP-ΔC2	Ligation	TPD1sp-nEYFP-ΔTPD1	zp541+zp1987	KpnI+XbaI	pSAT6-mRFP-N1
nEYFP-TPD1^{K135GR136G}	Ligation	35S:TPD1sp-GFP-ΔTPD1^{K135GR136G}	zp570+zp396	EcoRI+XbaI	nEYFP-TPD1
pCR2.1-mGFP5ER	TA cloning	pBIN Gal4–mGFP5*er*	zp1823+zp1824	XhoI+XbaI	pCR2.1
pEarleyGate303-mGFP5ER	Ligation	pCR2.1-mGFP5ER		XhoI+XbaI	pEarleyGate303
TPD1:mGFP5ER	LR Recombin ation	pENTR-TPD1:			pEarleyGate303 -mGFP5ER
pENTR-EMS1:	TOPO cloning	T28J14 (EMS1 BAC)	zp590+zp591		pENTR/D-TOPO
EMS1:mGFP5ER	LR Recombin ation	pENTR-EMS1:			pEarleyGate303 -mGFP5ER
pENTR-GFP	TOPO cloning	pEGAD	zp1174+zp1235		pENTR/D-TOPO
pENTR-2xGFP	Ligation	pEGAD	zp1194+zp1195	NcoI	pENTR-GFP
pENTR-EMS1:EMS	TOPO cloning	T28J14 (EMS1 BAC)	zp91+zp97		pENTR/D-TOPO
pENTR-EMS1:EMS1-2xGFP	Ligation	pENTR-2xGFP		AscI	pENTR-EMS1:EMS1
EMS1:EMS1-3xGFP	LR Recombin ation	pENTR-EMS1:EMS1-2xGFP			pGWB4
pENTR-ML1:	TOPO cloning	F17L22	zp543+zp544		pENTR/D-TOPO
ML1:mGFP5ER	LR Recombin ation	pENTR-ML1:			pEarleyGate303 -mGFP5ER
pENTR-ML1:TPD1	Ligation	U13545 (TPD1 cDNA)	zp541+zp542	KpnI+SacI	pENTR-ML1:
ML1:TPD1	LR Recombin	pENTR-ML1:TPD1			pGWB1
pENTR-ML1:ΔTPD1	Ligation	U13545 (TPD1 cDNA)	zp397+zp542	KpnI+SacI	pENTR-ML1:
ML1:ΔTPD1	LR Recombin	pENTR-ML1:ΔTPD1			pGWB1
pENTR-ML1:TPD1sp-GFP-ΔTPD1	Ligation	35S:TPD1sp-GFP-ΔTPD1	zp541+zp542	KpnI+SacI	pENTR-ML1:
ML1:TPD1sp-GFP-ΔTPD1	LR Recombin ation	pENTR-ML1:TPD1sp-GFP-ΔTPD1			pGWB1
pCR2.1-Barstar::Barstar	TA cloning	pABGCZ	zp1768+zp1769		pCR2.1
pEarleyGate303-Barstar	Ligation	pCR2.1-Barstar::Barstar		Nsi	pEarleyGate303
pCR2.1-Gateway Cassette B	TA cloning		zp1770+zp1771		pCR2.1

pEarleyGate303-Barstar-Gateway Cassette B		pCR2.1-Gateway Cassette B		BglII+XhoI	pEarleyGate303-Barstar
pEarleyGate303-Barstar-BARNASE	Ligation	pABGCZ	zp1909+zp1773	XhoI+XbaI	pEarleyGate303-Barstar-Gateway Cassette B
pENTR-SDS::SDS	TOPO cloning	F10B6 (SDS BAC)	zp1208+zp1282		pENTR/D-TOPO
SDS::SDS-BARNASE	LR Recombination	pENTR-SDS::SDS			pEarleyGate303-Barstar-
