# Supplementary material for: Control of Anther Cell Differentiation by the Small Protein Ligand TPD1 and Its Receptor EMS1 in Arabidopsis
Source: PLoS Genet. 2016 Aug 18;12(8):e1006147. doi: 10.1371/journal.pgen.1006147 (PMC4990239; doi:10.1371/journal.pgen.1006147)
Supplement: S2 Table — (PDF) [file pgen.1006147.s002.pdf]

Supplementary Table 2: Primers Used in This Study

Primers	Sequences (5' to 3')	Purpose
zp199	CACCCCGGGTGGGATACAACATGTTG	
zp200	GCTCTAGAGCGGGGTACCGTGCGTAGACGTCGAAGAACTAAA	
zp541	CACCGGTACCATGAACCGACGGCGACTTTTG	
zp396	GCTCTAGACTAAGCACATGTCACGAAGG	
zp397	CACCGGGGTACCATGGGTGAAAAGCTTAGAGATAATCTGGA	
zp415	CACCGGTACCATGGATTCGAAGAGTTTTGTGCTACTACTACTACTCTT CTGCTTCTTGTTCCTTCATCTCGTCTCCGTTGAAGCC	
zp393	CACCGGTACCGAGAAACTAATCTTGAAGATGAG	
zp394	CAGATTATCTCTAAGCGAACCCTCCATAGCAAAACTC	
zp395	GGTGAAAAGCTTAGAGATAATCTGGA	
zp396	GCTCTAGACTAAGCACATGTCACGAAGG	
zp47	ACTCTAGACAGCACATGTCACGAAGGC	
zp389	ACCTATTCTGCGACAAGAGTGGAGTTGGCGGCGATGGCCTCGGCGA AGACAGCACATGTCACGAAGGCGAC	
zp390	ACCTAACCACCTTCTGCGACAAGAGTGGAGTTGGCGGCGATGGCCT CGGCGAAGACAGCACATGTCACGAAGGCGAC	
zp871	GGGGTACCATGAGAATGGAACATATCTACAAAT	
zp872	GCTCTAGATCAGAAGCAAGAGACTGAAGC	
zp1829	GCTCTAGACTAAAAAGGCAAAGGCTTGCCGTTG	
zp1827	GCTCTAGACTAAGTACCAGGAGAGAGAAGCAGC	
zp1828	GCTCTAGACTATCCAGACATACACTGGTTCGTTATC	
zp1987	GCTCTAGACTAGACTCTTGGGTTTATCAACTTAGC	
zp1988	GCTCTAGACTACCTCGAAATTATGCATCCAGAC	
zp1912	ACGAACCAGTGTATGTCTGGAT	
zp1913	GACATACACTGGTTCGTACCGGAGGCTTCAACGGAG	
zp1919	ATGTCTGGATGCATAATTTCGA	
zp1920	TCGAAATTATGCATCCAGACATACCGGAGGCTTCAACGGAG	
zp1921	GATCCACATCAACTGCGGTTGG	
zp1922	CCAACCGCAGTTGATGTGGATCACCGGAGGCTTCAACGGAG	
zp1914	GGTTGGTTCAGCTCAGCTAAG	
zp1915	GCTGAGCTGAACCAACCACCGGAGGCTTCAACGGAG	
zp2006	GATCCACATCAACTCTGGTTGG	
zp2007	CCAACCAGAGTTGATGTGGATC	
zp977	GTTGAGCTCGCCGGCAGATTAGCCTTTTCAATTTCAG	
zp978	GCGACCGGTGCACCGGAGGCTTCAACGGAG	
zp570	GCCGAATTCATGAACCGACGGCGACTTTTG	
zp571	GAGCCCGGGCTAAGCACATGTCACGAAGG	
zp1029	CACCGGTACCGCGTGAGCAAGGGCGAGGAG	
zp2093	AGAGTCTTCGGTGGTATTCACTAC	
zp2094	GTAGTGAATACCACCGAAGACTCT	
zp1036	CACCGGTACCGCATGAACCGACGGCGACTTTTG	
zp52	CACCATGGCGTTTCTTACCGCATTGTTC	
zp73	TGGTCGACTCATATCTCCTTAAGAGCCTTC	
zp1064	CTCTCGAGACTTGTACAGCTCGTCCATGCCGAG	
zp1065	GCTGCACGCTGCCGCCCACGGCAACAATGGCGGAACTCGAAAAAG	
zp1066	GAGTTCCGCCATTGTTGCCGTGGGCGGCAGCGTGCAGC	
zp1070	TAGCCATGGGCGCGTTTCTTACCGCATTGTT	

zp1080	CAGGTACCTGGGATTTCTCCTGTGAAGTTG	
zp1071	GTACCGCGGGCCCTTCCAAGGCATTGCTTTCAGGG	
zp1072	GGTGGATCCTCAATGATCTGTAGCCTCGACAATATC	
zp1073	GATCTCGAGCTTGTCCAATGTTGAAACATCTCAGTC	
zp1074	CAGGTACCCAAACGAGAGAGTGAAGCTGGAATC	
zp1075	GATCTCGAGCTGTGAAGCAAAGAGATGATCCAGA	
zp1076	GATCTCGAGCTCAGTCTTTATACAGAGAAATCCATCAG	
zp1077	CAGGTAACCTCCAACTCCGCCTCTTTCTTTC	
zp1041	CAGGTAACATGATCTGTAGCCTCGACAATATC	
zp590	CACCAAGAAGCTGTAGCAGAGAGAACCAATGCAA	
zp591	GGGGTACCAAGCCGGCGTTCTTTTAGAGAAGGAG	
zp1174	CACCCTCGAGGGTACCGGGCGCGCCATGGTGAGCAAGGGCGAGGA	
zp1235	CGAGGCGCGCCATCTAGACTTGTACAGCTCGTCCATGCCGA	
zp1194	CACCCCATGGGAGGCGGTGGTGGAGCAGTGAGCAAGGGCGAGGAG	
zp1195	TCACCATGGCATCTCTTGTACAGCTCGTCCATGC	
zp91	CACC CAGAGAGAACCAATGCAACTC	
zp97	TATCTCCTTAAGAGCCTTCAACACATCAAGC	
zp979	CACCGCCGGCATGGCGTTTCTTACCGCATTG	
zp1163	CAATTCTCCGGGAACATTCCGCCTG	
zp1164	CAGGCGGAATGTTCCCGGAGAATTG	
zp272	TGGTACCTATCTCCTTAAGAGCCTTCAAC	
zp543	CACCGGCGTCAGAAAGAAACATAGG	
zp544	CAGGAGCTCTATGGTACCGATGATGATGGATGCCTATCAA	
zp542	CAGGAGCTCCTAAGCACATGTCACGAAGG	
zp397	CACCGGGGTACCATGGGTGAAAAGCTTAGAGATAATCTGGA	
zp1768	CCAATGCATTGGCGTATAACATAGTATCGAC	
zp1769	CCAATGCATATGGCAGCGCTGGCAGTC	
zp1770	GAAGATCTGGATCCGGCTTAC	
zp1771	GCTCTAGACTCGAGCTGTTCCACCACTTTGTAC	
zp1909	GCTCTAGAATGGCACAGGTTATCAACAC	
zp1931	CTCTACCATGTAGCCTGGCTTTTCTCCAATTCTC	
zp1932	GAGAATTGGAGAAAAGCCAGGCTACATGGTAGAG	
zp1933	ACGCTTGAAGACTCTTGGCTTTTCTCCAATTCTC	
zp1934	GAGAATTGGAGAAAAGCCAAGAGTCTTCAAGCGT	
zp1773	GCTCTAGAAGGATATCCTGATCCGTTGAC	
zp1208	CACCCCATCATTCTCGTCTCTCTCGCAC	
zp1282	CTTCTAGAGGTACCGCTGCCCAAGCAACCAGTCCAG	
zp1210	CACCGGATTATAATAATGTGTAGACATTGTAGG	
zp1211	CATCTAGACTCGAGGGTACCTCTAATTAGATACTATATTGTTTGTAC	
zp1184	ACAGAAGTACAAACAATATAGTATC	See supplementary
zp2432	GCTCTAGACTACCTCGAAATTATGCATCCAGAC	Table 1 for the
zp2433	AGGAATTCATAACGAACCAGTGTATGTCTGG	usage of above
zp1185	TTCAATAGATCTGTCATAAGTATTG	primers
zp53	ATGAACCGACGGCGACTTTTGG	RT-PCR for
zp1030	TCAGGCCGCTGCCGCAGCGGC	TPD1sp-ΔTPD1-
zp2161	CACAAGCCCTCGGCTGGGTG	Real-time PCR for
zp2162	GCTTCACGCCATGTTCGTCC	*BARNASE*
zp1448	TACCTAAACCGACGAACA	Real-time PCR for
zp1449	ATGCCAATAAATG GAGAC	*A6*
zp1196	TGCCCTCCAAGCAACTAACAA	Real-time PCR for

zp1197	GCTGCTCGAAGAGCGTTACAT	*A9*
zp853	GTTGGGATGAACCAGAAGGA	Real-time PCR for
zp854	GAGGAGCCTCGGTAAGAAGA	*ACTIN2*
ZP851	CGTCTCCAGGATCGAGGAAT	Real-time PCR for
ZP852	GGAGATGGGAAAGCTGAGAG	*ATA7*
