# Supplementary figures and images for: Control of Anther Cell Differentiation by the Small Protein Ligand TPD1 and Its Receptor EMS1 in Arabidopsis
Source: PLoS Genet. 2016 Aug 18;12(8):e1006147. doi: 10.1371/journal.pgen.1006147 (PMC4990239; doi:10.1371/journal.pgen.1006147)

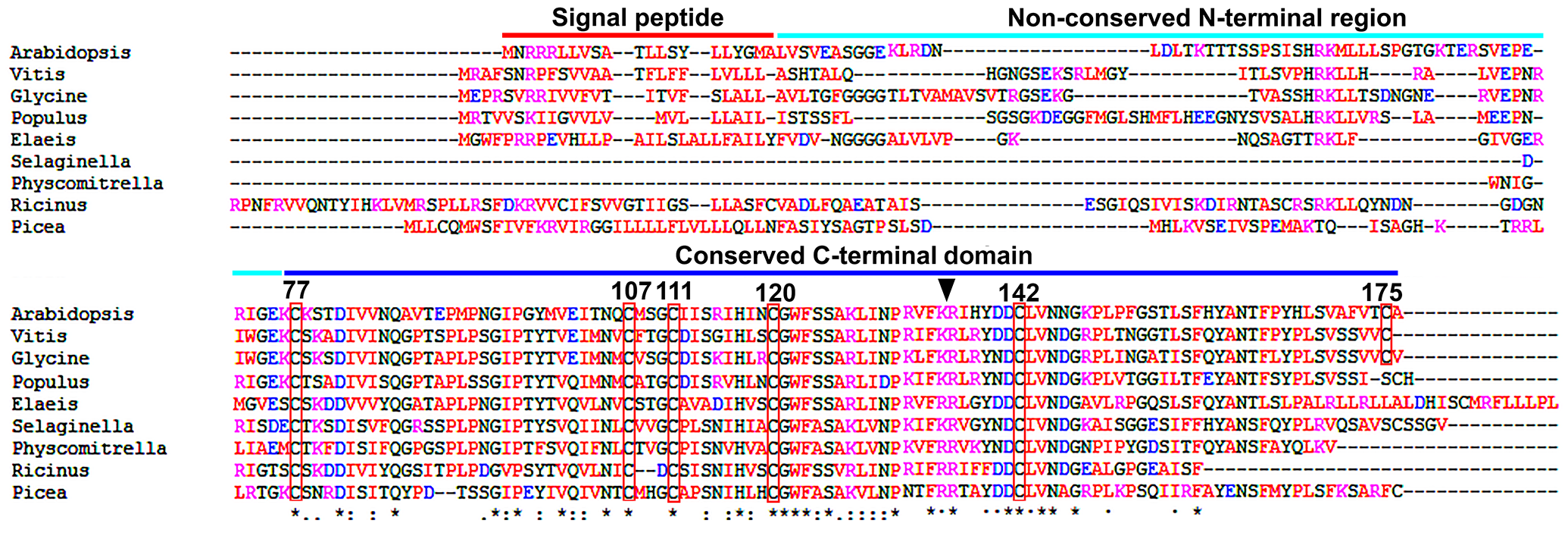

Supplement: S1 Fig — ClustalW2 was used for sequence alignment. Sequences were retrieved from the NCBI database. Numbers and frames indicate the positions of cysteine residues. The domain definition is based on the Arabidopsis TPD1 sequence. Red line: the putative signal peptide, Cyan line: the non-conserved N-terminal region, Blue line: the conserved C-terminal domain. Arrowhead indicates the putative dibasic cleavage site. (TIF) [file pgen.1006147.s003.tif]

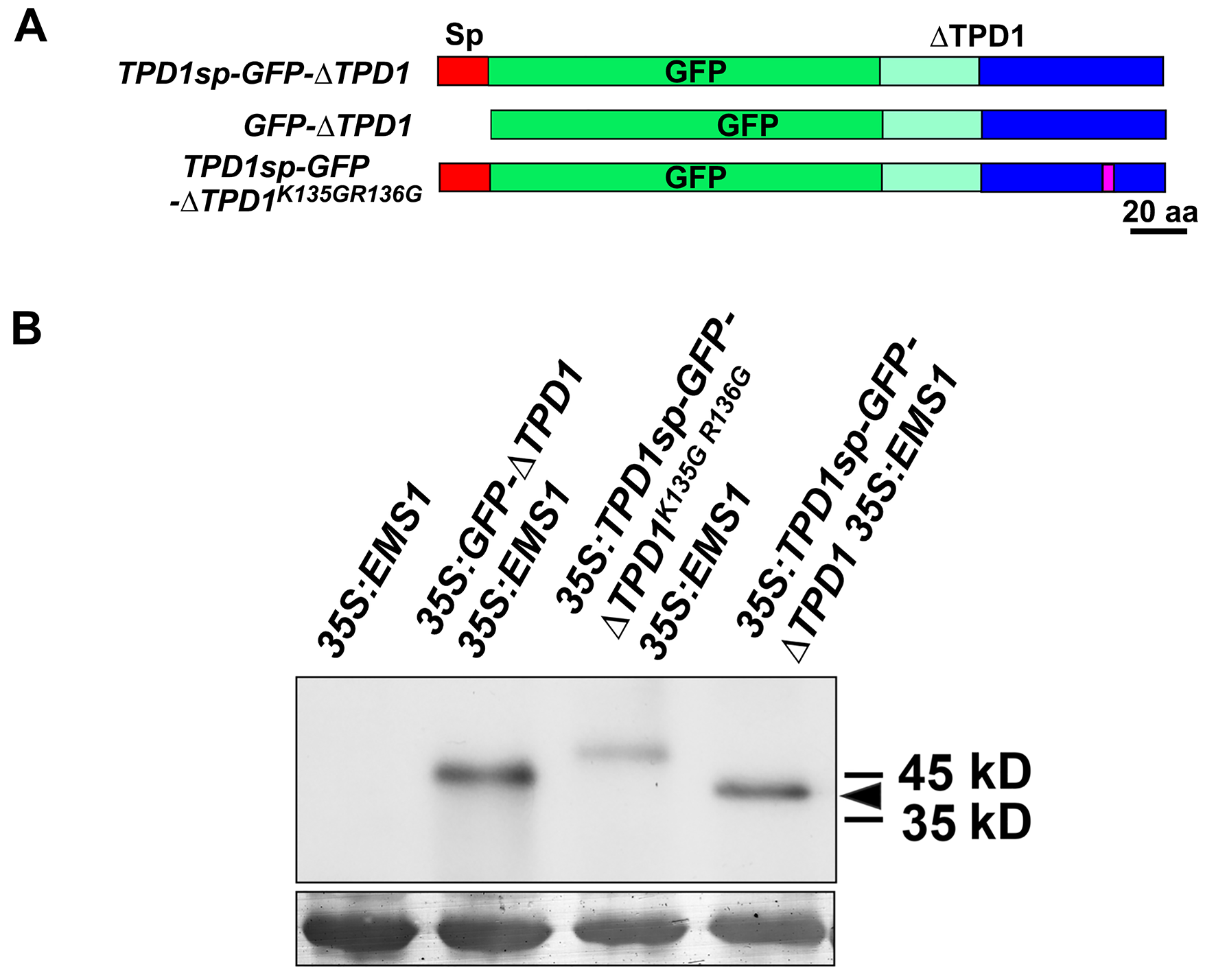

Supplement: S2 Fig — (A) Schematic diagrams showing the structures of the TPD1sp-GFP-ΔTPD1, GFP-ΔTPD1, and TPD1sp-GFP-ΔTPD1K135G R136G constructs. Red bar: the TPD1 putative signal peptide (Sp), Green bar: GFP, Cyan bar: the non-conserved N-terminal region, Blue bar: the conserved C-terminal domain, ΔTPD1: TPD1 without the putative signal peptide, and Pink line: K135GR135G mutations. (B) Western blotting was used to examine the processing of GFP-fused TPD1 proteins extracted from transfected 35S:EMS1 leaf protoplasts. 35S:EMS1 shows no band; 35S:GFP-ΔTPD1 35S:EMS1 exhibits a 45-kD band; 35S:TPD1sp-GFP-ΔTPD1K135G R136G 35S:EMS1 exhibits a 48-kD band; and 35S:TPD1sp-GFP-ΔTPD1 35S:EMS1 exhibits a 41-kD band (arrow). (TIF) [file pgen.1006147.s004.tif]

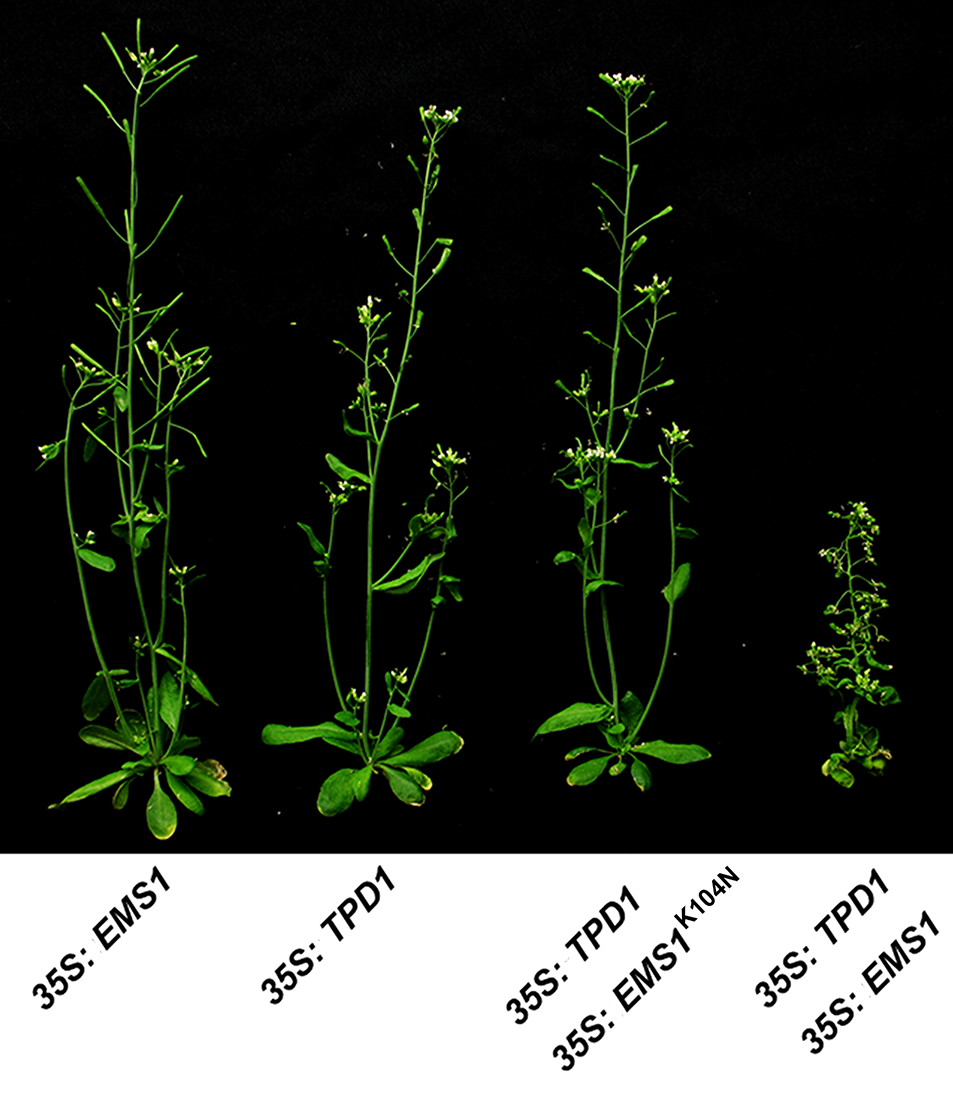

Supplement: S3 Fig — To examine the importance of the interaction of TPD1 with EMS1 in the first three LRRs, 35S:EMS1, 35S:TPD1, 35S:TPD1 35S:EMS1K104N, and 35S:TPD1 35S:EMS1 plants were generated and analyzed. The 35S:EMS1 plant appeared similar to the wild-type plant. The 35S:TPD1 plant produced short and wide siliques, but was nearly normal in stature. The 35S:TPD1 35S:EMS1 plant was dwarf and had twisted leaves, stem, inflorescences, and siliques. In contrast, the 35S:TPD1 35S:EMS1K104N plant resembled the 35S:TPD1 plant. (TIF) [file pgen.1006147.s005.tif]

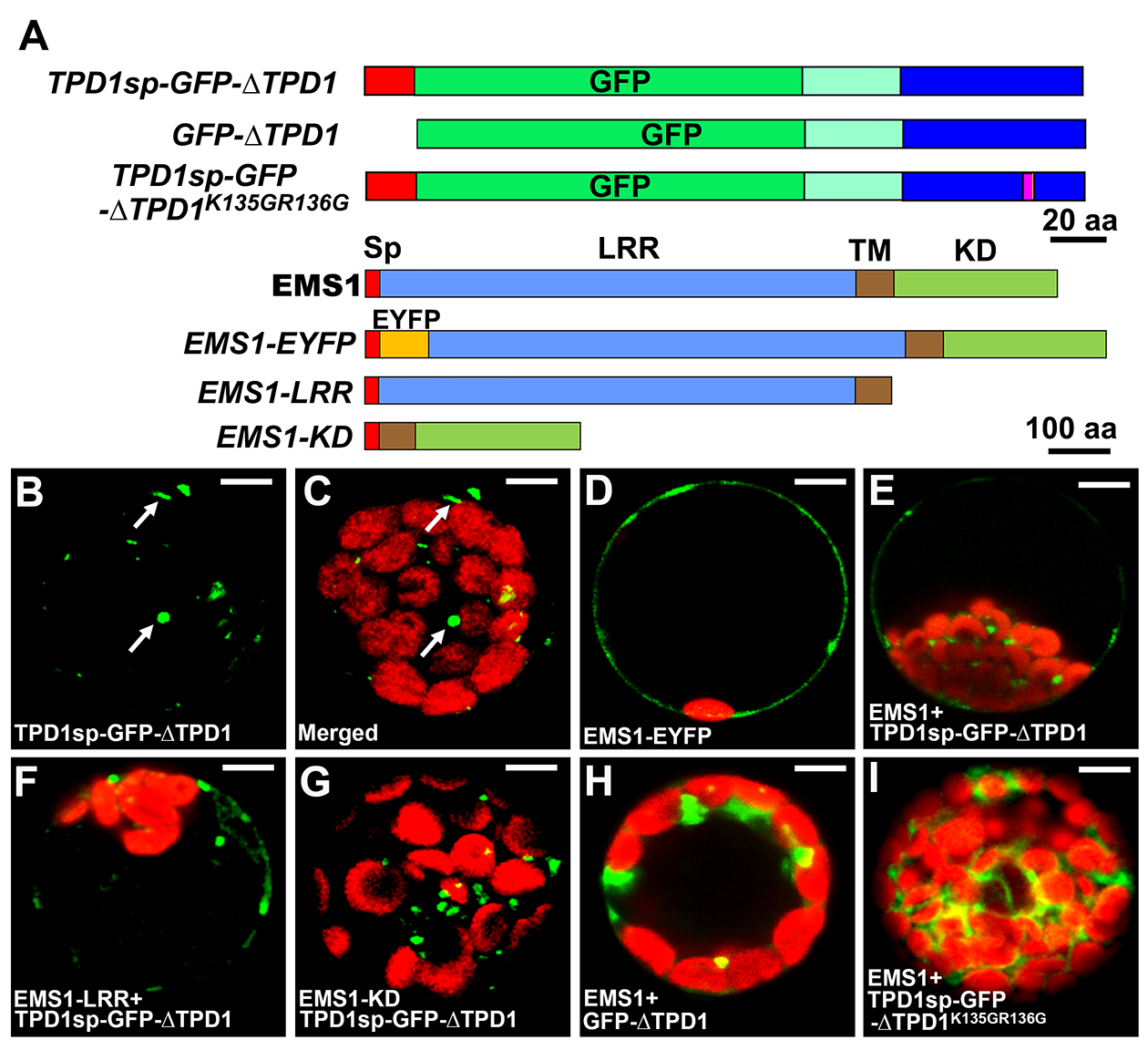

Supplement: S4 Fig — (A) Schematic diagrams showing the truncated and mutated versions of TPD1 and EMS1. For TPD1 constructs, Red bar: the TPD1 putative signal peptide (Sp), Green bar: GFP, Cyan bar: the non-conserved N-terminal region, Blue bar: the conserved C-terminal domain, ΔTPD1: TPD1 without the putative signal peptide, and Pink line: K135GR135G mutations. For EMS1 constructs, Red bar: the EMS1 putative signal peptide (Sp), Dodger blue bar: leucine-rich repeat (LRR), Brown bar: the transmembrane domain (TM), Olive green bar: kinase domain (KD), and Yellow bar: EYFP. (B, C) Confocal images showing TPD1sp-GFP-ΔTPD1 inside the leaf protoplast [B, GFP signal; C, GFP merged with chlorophyll autofluorescence (red)]. Arrows indicate GFP signals in trafficking vesicle-like compartments. (D-I) Merged confocal images. (D) Full-length EMS1-EYFP at the plasma membrane. (E, F) TPD1sp-GFP-ΔTPD1 at the plasma membrane in the presence of full-length EMS1 (E) and the EMS1 LRR domain (F). (G) TPD1sp-GFP-ΔTPD1 is not observed at the plasma membrane in the presence of the EMS1 kinase domain (KD). (H, I) GFP-ΔTPD1 (H) and TPD1sp-GFP-ΔTPD1K135G R136G (I) were not found at the plasma membrane or in trafficking vesicle-like compartments, regardless of the presence of EMS1. Scale bars, 10 μm. (TIF) [file pgen.1006147.s006.tif]

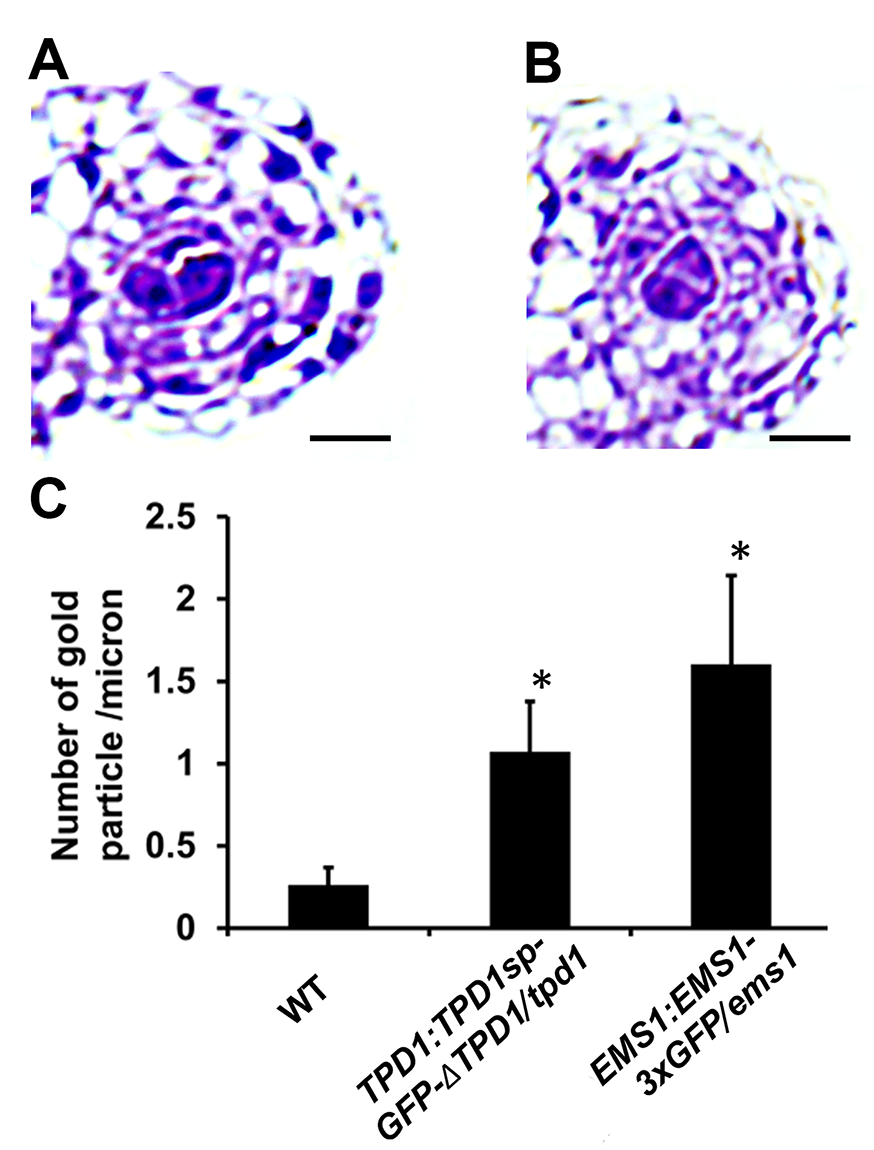

Supplement: S6 Fig — (A, B) Semi-thin sections showing the early-stage-5 TPD1:TPD1sp-GFP-ΔTPD1/tpd1 (A) and EMS1:EMS1-3xGFP/ems1 (B) anthers used for EM-immunolabeling (Fig 6T and 6U). Scale bars, 10 μm. (C) Statistics for the numbers of gold particles per micron at the plasma membrane and between cells. The length of the plasma membrane was measured using the ImageJ software. Stars indicate that the numbers of gold particles in TPD1:TPD1sp-GFP-ΔTPD1/tpd1 and EMS1:EMS1-3xGFP/ems1 anthers are significantly higher than that in wild-type anthers (P<0.01). (TIF) [file pgen.1006147.s008.tif]

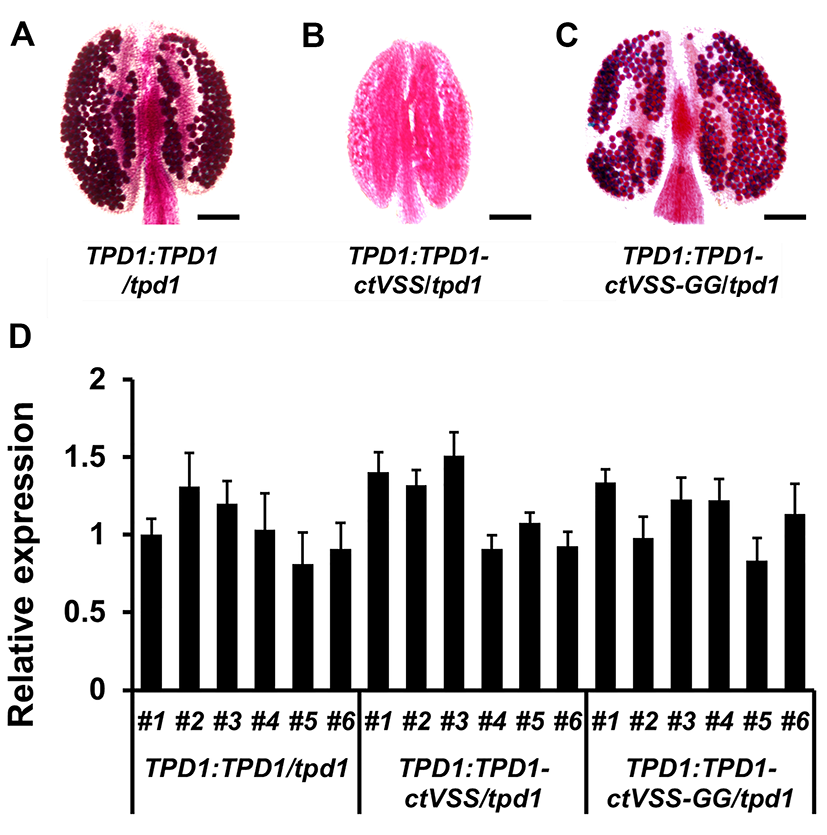

Supplement: S7 Fig — (A-C) Pollen viability was tested by Alexander pollen staining: (A) The TPD1:TPD1/tpd1 anther exhibits functional pollen grains, (B) no pollen is observed in the TPD1:TPD1-ctVSS/tpd1 anther, and (C) normal pollen grains are seen in the TPD1:TPD1-ctVSS-GG/tpd1 anther. Scale bars, 50 μm. (D) qRT-PCR was used to examine TPD1 expression in anthers of six plants each from the TPD1:TPD1/tpd1 complemented lines, TPD1:TPD1-ctVSS/tpd1 sterile lines, and TPD1:TPD1-ctVSS-GG/tpd1 complemented lines. The results showed that the expression levels of TPD1 were similar in all tested plants. (TIF) [file pgen.1006147.s009.tif]

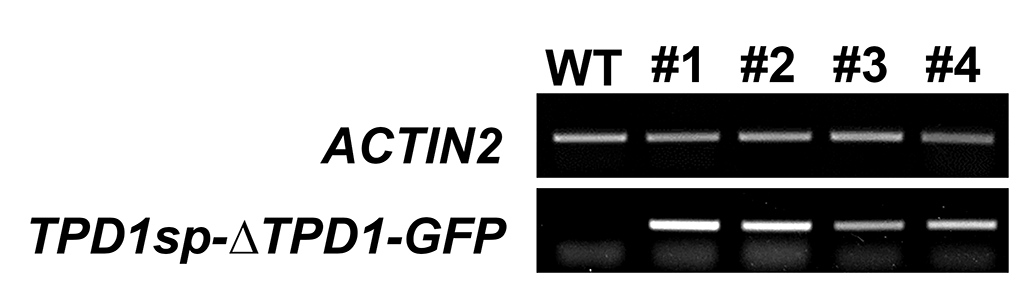

Supplement: S8 Fig — RT-PCR was used to examine the expression of the TPD1sp-ΔTPD1-GFP fusion gene in anthers. Four lines of TPD1:TPD1sp-ΔTPD1-GFP/tpd1 plants were tested. TPD1sp-ΔTPD1-GFP expression was detected in all four transgenic lines but not in wild-type (WT) plants. The ACTIN2 gene was used as the internal standard. (TIF) [file pgen.1006147.s010.tif]

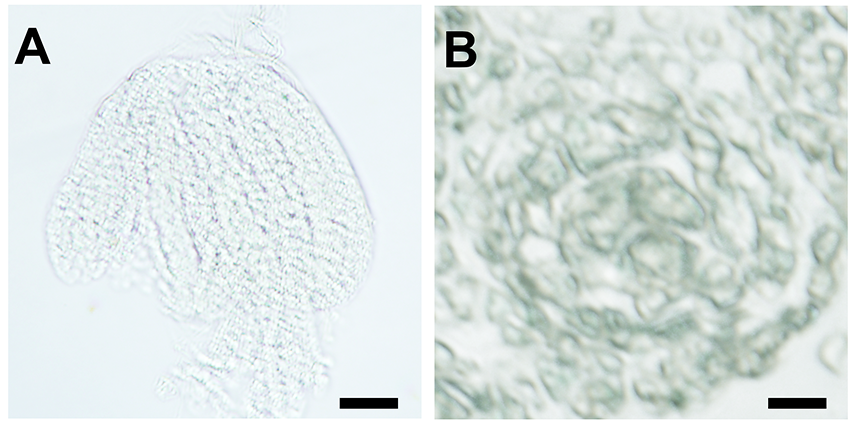

Supplement: S9 Fig — GUS staining was used to examine the expression of TPD1sp-ΔTPD-GUS in anthers. Twenty TPD1:TPD1sp-ΔTPD-GUS plants were analyzed, but none showed any positive GUS signal. (A) A stage-5 anther showing no GUS signal. (B) A transverse section of a stage-5 anther showing no GUS signal. (TIF) [file pgen.1006147.s011.tif]
